# Emergence of social inequality in the spatial harvesting of renewable public goods

**DOI:** 10.1371/journal.pcbi.1007483

**Published:** 2020-01-08

**Authors:** Jaideep Joshi, Åke Brännström, Ulf Dieckmann

**Affiliations:** 1 Centre for Ecological Sciences, Indian Institute of Science, Bengaluru, India; 2 Evolution and Ecology Program, International Institute for Applied Systems Analysis, Laxenburg, Austria; 3 Department of Mathematics and Mathematical Statistics, Umeå University, Umeå, Sweden; 4 Department of Evolutionary Studies of Biosystems, The Graduate University for Advanced Studies (Sokendai), Hayama, Kanagawa, Japan; University of Chicago, UNITED STATES

## Abstract

Spatially extended ecological public goods, such as forests, grasslands, and fish stocks, are at risk of being overexploited by selfish consumers–a phenomenon widely recognized as the ‘tragedy of the commons.’ The interplay of spatial and ecological dimensions introduces new features absent in non-spatial ecological contexts, such as consumer mobility, local information availability, and strategy evolution through social learning in neighborhoods. It is unclear how these features interact to influence the harvesting and dispersal strategies of consumers. To answer these questions, we develop and analyze an individual-based, spatially structured, eco-evolutionary model with explicit resource dynamics. We report the following findings. (1) When harvesting efficiency is low, consumers evolve a sedentary consumption strategy, through which the resource is harvested sustainably, but with harvesting rates far below their maximum sustainable value. (2) As harvesting efficiency increases, consumers adopt a mobile ‘consume-and-disperse’ strategy, which is sustainable, equitable, and gives maximum sustainable yield. (3) A further increase in harvesting efficiency leads to large-scale overexploitation. (4) If costs of dispersal are significant, increased harvesting efficiency also leads to social inequality between frugal sedentary consumers and overexploitative mobile consumers. Whereas overexploitation can occur without social inequality, social inequality always leads to overexploitation. Thus, we identify four conditions that–while being characteristic of technological progress in modern societies–risk social inequality and overexploitation: high harvesting efficiency, moderately low costs of dispersal, high consumer density, and the tendency of consumers to adopt new strategies rapidly. We also show how access to global information–another feature widespread in modern societies–helps mitigate these risks.

## Author summary

Throughout history, humans have shaped ecological landscapes, which in turn have influenced human behavior. This mutual dependence is epitomized when human consumers harvest a spatially extended renewable resource. Simple models predict that, when multiple consumers harvest a shared resource, each is tempted to harvest faster than his/her peers, putting the resource at risk of overexploitation. It is unclear, however, how the interplay among resource productivity, consumer mobility, and social learning in spatial ecological public goods games influences evolved consumer behavior. Here, using an individual-based, spatially structured, eco-evolutionary model of consumers and a resource, we find that increasing resource productivity initially promotes efficient resource use by enabling mobile consumption strategies, but eventually leads to inequality and overexploitation, as overexploitative mobile consumers coexist with frugal sedentary consumers. When consumers are impatient (i.e., eager to imitate successful strategies) or myopic (i.e., unaware of conditions outside of their neighborhoods), inequality and overexploitation tend to aggravate.

## Introduction

A public good that is freely accessible to everyone, while limited in quantity, can be used optimally only if consumers cooperate in using no more than their fair share. However, those who consume more than their fair share obtain a greater benefit. This creates an incentive to overexploit the public good, which threatens to reduce resource availability, leaving everyone worse off. This social dilemma is called the ‘tragedy of the commons’ [1, 2]. The ubiquity of such social dilemmas has led to widespread interest in exploring mechanisms that can support cooperation, not only in evolutionary biology but also in economics, cognitive sciences, and social sciences [3–7]. Indeed, research into mechanisms capable of preventing social dilemmas can help inform important policy decisions, ranging from fisheries regulations and forest management to treaty negotiations on the global commons, such as ozone-layer protection and climate-change mitigation [8–17].

Owing to the combined efforts of many scientists, several mechanisms that promote cooperation have been identified (e.g., [18]). Kin selection [19], in which cooperative acts are directed towards genetic relatives, and direct reciprocity [20–24], in which consumers cooperate with others returning their help, are two key mechanisms that promote cooperation. For humans and other animals with sufficient cognitive capabilities, indirect reciprocity, in which consumers cooperate with those who have a reputation for cooperating [25–27], is also an important mechanism supporting cooperation. Furthermore, combinations of positive and negative incentives, such as rewarding cooperators and punishing defectors, can be powerful means of promoting cooperation [28–31]. Nonlinear effects leading to a decreasing marginal benefit of cooperation have been shown to promote the coexistence of cooperators and defectors [32, 33]. Studies of the aforementioned mechanisms are mostly stylized, often based on simple mathematical models and occasionally on experiments.

Most real socio-ecological systems are spatially structured. Spatial structure is thought to promote cooperation, as it exposes defectors to the negative local consequences of their selfish acts and allows clusters of cooperators to form, generating positive assortment [34–36]. However, space also allows mobility. Mobility can hinder cooperation by allowing defectors to escape the negative local consequences of their selfish acts [37]. Indeed, several studies have considered the effects of fixed mobility and found that cooperation can be sustained when mobility is either low [38] or dependent on local conditions [39,40]. If mobility incurs no cost, defectors may invariably evolve high mobility and undermine cooperation. When dispersal is costly, an evolutionary interplay between mobility and cooperation may occur, but thus far only a handful of studies have considered the joint evolution of costly mobility and cooperation [41–45]. A recent study by Mullon et al. [46] found that when dispersal and cooperation coevolve, two coexisting strategies can spontaneously emerge: one benevolent and sessile, the other self-serving and dispersing.

In socio-ecological settings, the ecological dynamics of the resource play a crucial role in determining the associated evolutionary dynamics of consumer strategies. First, interactions between consumers are often mediated through the resource, i.e., consumers do not directly interact with other consumers, but respond to resource changes caused by other consumers. Second, limited resource availability may lead to the evolution of density-dependent strategies, since per capita resource extraction is expected to decline with local consumption density. Third, ecological public goods, such as renewable resources, have their own ecological timescale of resource replenishment. Most studies assume that evolution is a slow process. However, memetic evolution through social learning is not biologically constrained, and can occur on much faster timescales. Evolutionary outcomes may be dramatically different depending on whether evolution is slow or fast [47]. Furthermore, spatial structure may prevent consumers from having full knowledge about other consumers and the environment, because information about far-off conditions may not reach them. Local information may lead to local selection, which typically benefits defectors. Despite these important gaps, only a few studies have so far considered ecological public goods (e.g., [2, 48,49]), and even fewer studies have explicitly modelled a renewable resource (e.g., [50]). Here, we move beyond the aforementioned studies, in particular the pioneering work by Parvinen [45] and Mullon et al. [46], by investigating the implications of explicit resource dynamics, fast evolution, and incomplete information on the evolution of cooperation. Using a socio-ecological model of public-goods utilization, also referred to as the common-pool-resource model in economics, we investigate for the first time the coevolution of resource harvesting and dispersal strategies in realistic socio-ecological settings.

## Methods

**Fig 1:**
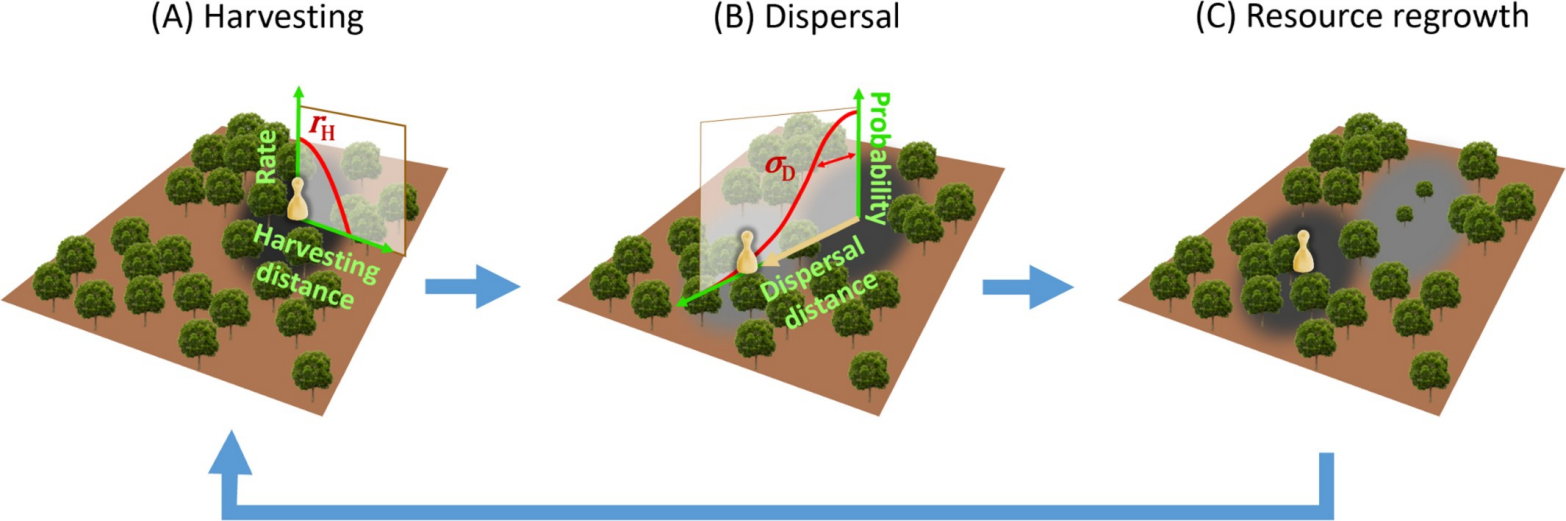
Schematic representation of our model’s processes and evolving traits. For the sake of illustration, only a single consumer is shown. (A) Consumers harvest the resource in their neighborhoods at a rate that declines with the distance from their locations. (B) When the local resource density falls below a threshold, consumers disperse to other locations at distances randomly drawn from a normal distribution. (C) Once consumers have dispersed, the resource densities around their original location can rebound.

We model consumers harvesting a resource in a continuous two-dimensional square-sharped space of dimension *L*×*L* with periodic boundaries. We assume that resource growth is logistic. Each consumer *i* exploits the resource in his/her neighborhood (see below) at a harvesting rate *r*_H,*i*_. When the local resource density falls below a threshold, consumers disperse in random directions to new locations drawn from a normal distribution centered on their current locations, with a standard deviation, or dispersal radius, of *σ*_D,*i*_. Each consumer *i* is thus characterized by two quantitative traits, *r*_H,*i*_ and *σ*_D,*i*_, defining his/her resource-consumption strategy (Fig 1). Consumers occasionally imitate the resource-consumption strategies of those consumers in their neighborhood (see below) who acquire a higher profit from their resource consumption.

Details of the resultant resource-consumer dynamics and strategy dynamics are specified below. Table 1 lists all model parameters and S1 Table provides a glossary of key terms.

**Table 1:** Model parameters.

Parameter	Symbol	Default value
*Resource dynamics* Intrinsic resource growth rate	*r*	1
Carrying-capacity density	*K*	1
*Consumer dynamics* Harvesting radius	*σ*_H_	1
Harvesting rate of consumer *i*	*r*_H,*i*_	Evolving trait
Dispersal radius of consumer *i*	*σ*_D,*i*_	Evolving trait
Maximum dispersal rate	*r*_D,max_	500
Resource-density threshold for dispersal	*R*_T_	0.3
System size	*L*	56.25
Consumption density	*ρ*	50.8%
*Consumer payoffs* Unit benefit of harvesting	*b*_H_	0.059
Unit cost of harvesting	*c*_H_	0.68
Unit cost of dispersal	*c*_D_	1
Duration over which payoffs are averaged	*T*	0.4
*Strategy dynamics* Imitation radius	*σ*_I_	∞
Imitation rate	*r*_I_	0.1
Imitation error in harvesting rate	*μ*_H_	0.1
Imitation error in dispersal radius	*μ*_D_	0.05

### Resource dynamics

The resource density *R* at each location (*x*,*y*) grows logistically, with the harvesting-rate density *H* depending on all consumers exploiting that location,

dR(x,y)/dt=rR(x,y)(1−R(x,y)/K)−H(x,y), (1)

where *r* is the resource’s intrinsic growth rate and *K* is its carrying-capacity density, which we assume to be spatially uniform, i.e., there is no intrinsic spatial heterogeneity.

### Consumer dynamics

The initial locations of consumers are drawn from a uniform distribution over the entire space. The total number *N* of consumers remains constant over time, as consumers neither die nor reproduce.

Consumers harvest the resource in their neighborhoods according to an exploitation kernel *E* given by a quadratic function of spatial distance $d=\sqrt{\Delta x^{2}+\Delta y^{2}}$. Here, Δ*x* = *x*−*x*_*i*_ and Δ*y* = *y*−*y*_*i*_ are the differences in *x* and *y*, respectively, between the location (*x*_*i*_,*y*_*i*_) of the consumer and the location (*x*,*y*) of harvesting. Specifically,

$$E(d)=\left\{ \begin{array}{cc} 1-d^{2}/\sigma_{H}^{2} & \mathrm{if}\;d<\sigma_{H},\\ 0 & \mathrm{otherwise}, \end{array}\right.$$ (2)

where *σ*_H_ is the harvesting radius within which consumers extract the resource from their neighborhoods. On this basis, we measure the consumption density as the ratio of harvested area to total area, $\rho =\pi \sigma_{H}^{2}N/L^{2}$, rather than the typically used measure *N*/*L*^2^. This allows us to measure density as a dimensionless quantity independent of the length unit. The resource extraction-rate density of consumer *i* at location (*x*,*y*) is

$$H_{i}(x,y)=r_{H,i}R(x,y)E(x-x_{i},{y-y}_{i}).$$ (3)

Describing *H*_*i*_ as being proportional to *R* corresponds to a so-called Holling type-I functional response [51]. Assuming that the system size *L* is much larger than the harvesting radius *σ*_H_ (fulfilling at least *L*>2*σ*_H_), the total extraction rate of consumer *i* is

$$R_{H,i}=\int_{0}^{L}\int_{0}^{L}H_{i}(x,y)\;dxdy,$$ (4)

and the extraction-rate density at location (*x*,*y*) by all *N* consumers together is

$$H(x,y)=\sum_{i=1}^{N}H_{i}(x,y).$$ (5)

When local conditions become adverse, in terms of the resource density dropping below a threshold *R*_T_, consumers disperse to another location. The dispersal rate of consumer *i* is thus given by

$$r_{D,i}=r_{D,\max}S_{D}(R(x_{i},y_{i})-R_{T}).$$ (6)

where *r*_D,max_ is the maximal dispersal rate (i.e., the maximum number of dispersal events per time unit) and *S*_D_(Δ*R*) denotes a unit step function that switches from 1 for Δ*R*<0 to 0 for Δ*R*≥0. The distance to their new location is the absolute value of a random number drawn from a dispersal kernel given by a normal distribution with zero mean and a standard deviation, or dispersal radius, of *σ*_D,*i*_. Therefore, the mean dispersal distance of consumer *i* is $\sqrt{2/\pi }\sigma_{D,i}$. Dispersal is undirected, i.e., the direction of dispersal is a random number drawn from a uniform distribution over [0,2*π*). Since the boundaries are periodic, consumers that disperse across a boundary reappear on the other side.

### Consumer payoffs

Harvesting yields a benefit *b*_H_*R*_H,*i*_ and incurs a cost $c_{H}r_{H,i}^{2}$ per unit time, while dispersal incurs a cost *c*_D_ per unit length. We use a linear benefit and a quadratic cost to describe a diminishing marginal utility of the resource. The payoff of consumer *i*, averaged over a duration *T* ending at time *t*, is thus given by

$$V_{i}=\frac{1}{T}(\int_{t-T}^{t}\left(b_{H}R_{H,i}-c_{H}r_{H,i}^{2}\right)\;dt-c_{D}l_{D,i}),$$ (7)

where *l*_D,*i*_ is the cumulative distance consumer *i* has dispersed during time *T* (i.e., the actual distance traversed by the consumer, rather than merely the displacement between the consumer’s old and new locations).

### Strategy dynamics

To account for strategy evolution by social learning, we assume that consumers occasionally imitate the resource-consumption strategy, i.e., the harvesting rate and the dispersal radius, of other consumers with higher payoff. Specifically, at rate *r*_I_ each consumer *i* randomly chooses another consumer as a potential role model, with consumer *j* at location (*x*_*j*_,*y*_*j*_) being chosen with probability

$$p_{I,ij}\propto \exp(-\frac{{\left(x_{i}-x_{j}\right)}^{2}+{\left(y_{i}-y_{j}\right)}^{2}}{2\sigma_{I}^{2}}),$$ (8)

where *σ*_I_ is the imitation radius, and the proportionality constant is chosen to normalize the probabilities such that $\sum_{j=1,j\neq i}^{N}p_{I,ij}=1$ for all *i*. After a consumer *j* is chosen, the focal consumer *i* imitates the resource-consumption strategy of consumer *j* if *V*_*j*_ exceeds *V*_*i*_.

To describe implementation errors in strategy imitation, we add, upon each imitation event, normally distributed increments to both trait values, using standard deviations *μ*_H_ and *μ*_D_, respectively, for the harvesting rate and the dispersal radius. If a trait value becomes negative through such an addition, it is set to 0. As robustness checks, we also consider perception errors, through which consumers make errors in judging the payoffs of other consumers during imitation (S8 Fig) or in judging the local resource levels during dispersal (S9 Fig).

### Model parameters

Without loss of generality, we reduce the number of parameters by carefully choosing the units in our model (S1 Appendix). A key model parameter is the consumption density *ρ*, and our model has two pairs of additional essential parameters. The first pair are the payoff parameters *b*_H_ and *c*_H_ that determine the efficiency *b*_H_/*c*_H_ and utility $b_{H}R_{H,i}-c_{H}r_{H,i}^{2}$ of consumption. The second pair are the imitation parameters *r*_I_ and *σ*_I_ that determine the temporal and spatial scales of social learning.

The imitation rate *r*_I_ defines the temporal scale of strategy evolution: consumers are ‘impatient’ if their imitation rate is much larger than the intrinsic rate of resource growth; such consumers tend to imitate other consumers even before the consequences of the other consumers’ consumption strategies on the resource become apparent. The imitation radius *σ*_I_ defines the spatial scale of strategy evolution: consumers are ‘myopic’ if their imitation radius is so small that they can imitate only a few neighbors, whereas a very large or infinite imitation radius means that consumers possess global information and can thus imitate any other consumer in the population. In this way, we model essential characteristics of human behavior to make our model realistic, while leaving out other confounding factors to keep our model minimal.

### Technical parameters and model runs

We use an agent-based implementation of our model and run it from an initial condition until the evolved strategies have stabilized. To minimize stochasticity in the evolutionary dynamics, we simulate the largest computationally feasible system: we use *N* = 512 consumers in a system of size *L* = 56.25, corresponding to a consumption density of *ρ* = 50.8%, with space discretized into 450×450 = 202,500 cells. A model run of 750,000 time steps takes about 4 minutes on an NVIDIA Tesla K20 Graphics Processing Unit (GPU). The code developed for running our model is available at https://github.com/jaideep777/Consumer-resource-system and at Zenodo (DOI: 10.5281/zenodo.3601735). S1 Video shows a representative model run of the combined resource-consumer dynamics.

### Baseline harvesting and resource-consumption rates

We compare the evolved strategies–i.e., harvesting rates and dispersal radii–and emergent resource extraction rates with their expected values in two baseline scenarios. Each baseline scenario is calculated assuming that all consumers adopt the same strategy. In the first baseline scenario, consumers maximize yield by adopting harvesting rates and dispersal rates that maximize per-capita resource extraction (as defined by Eq 4). In the second baseline scenario, consumers maximize profit by adopting harvesting rates and dispersal rates that maximize payoff (as defined by Eq 7). Both strategies are calculated numerically for mobile consumers and analytically for sedentary consumers (S2 Appendix). The yield-maximizing strategies for sedentary and mobile consumers are consistently colored blue and green, respectively, in Figs 4–7.

The analytical calculation of the sedentary yield-maximizing strategy is possible in the limit of vanishing overlap among the harvesting kernels of consumers. Notice that this simplification does not affect any of our results, as the sedentary yield-maximizing strategy is used only as a reference for determining the color scales in Figs 4–7. Nonetheless, it is reassuring that these analytical calculations are reasonably accurate for consumption densities of up to 50% (S13 Fig).

## Results

To explore the social dynamics of harvesting and dispersal in ecologically realistic environments, we examine our model for a wide range of parameter combinations corresponding to diverse socio-ecological settings. Specifically, we investigate the impacts of consumption density, of unit costs and benefits of harvesting and dispersal, and of imitation rate and radius on the evolved resource-consumption strategies. Furthermore, we document the robustness of our results to changes in other model parameters and model structure.

### Sedentary and mobile strategies emerge spontaneously

**Fig 2:**
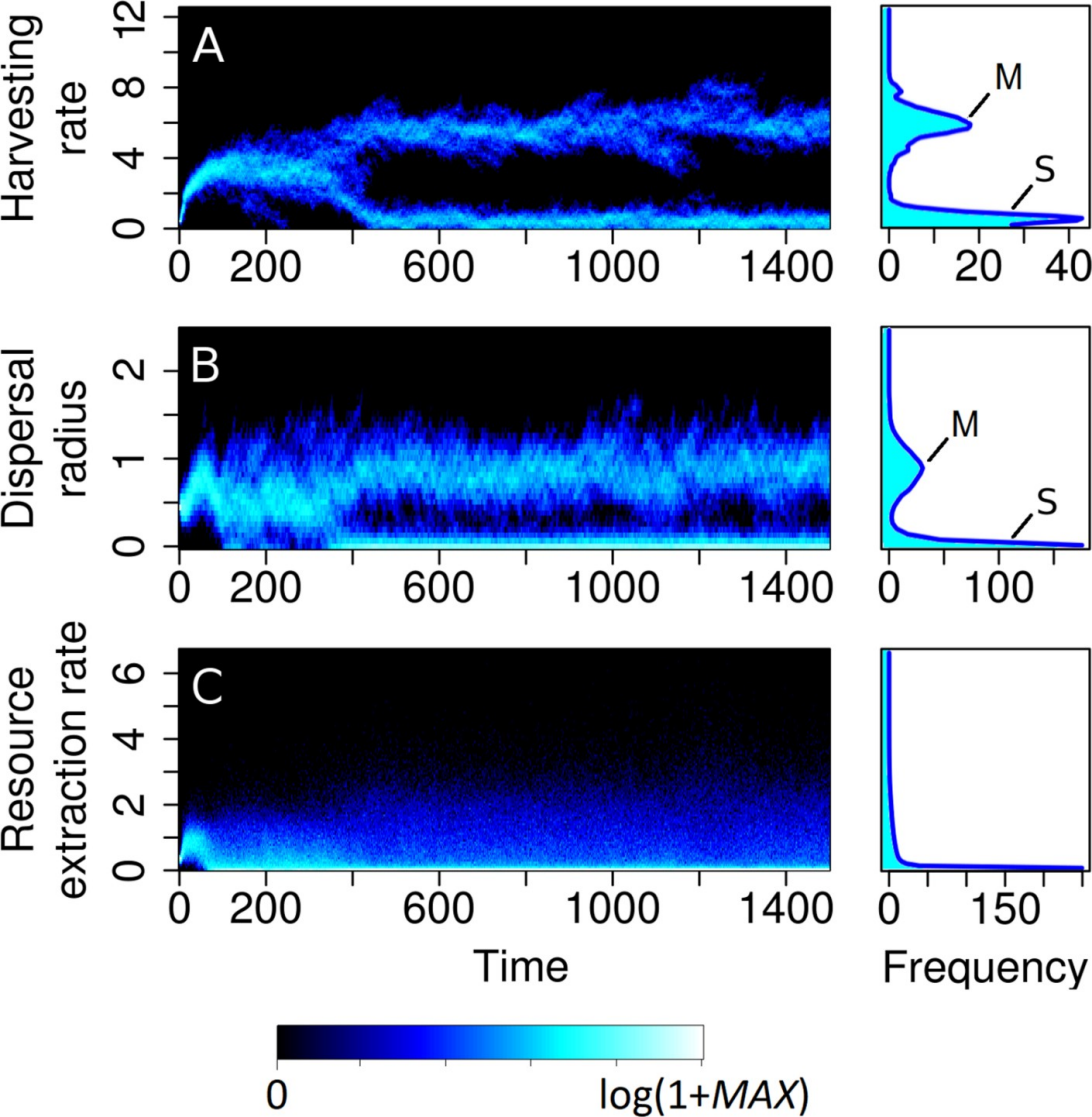
Sedentary and mobile resource-consumption strategies emerge spontaneously. Changes over time (measured in units of the reciprocal of the imitation rate) of the population distributions of (A) harvesting rates, (B) dispersal radii, and (C) per capita resource extraction rates, shown alongside the time-averaged population distributions of these quantities (averaged over the last 1,000 time units so as to exclude the initial transient, with the frequencies in the separate bins adding up to the number *N* of consumers). Brighter colors indicate higher frequencies, using a nonlinear color scale. The population starts with all consumers having identical strategies. As time progresses, social learning leads to the spontaneous emergence of two distinct strategies: a frugal sedentary strategy (labeled ‘S’) with a low harvesting rate and near-zero dispersal radius, and an overexploitative mobile strategy (labeled ‘M’) with a high harvesting rate and large dispersal radius. Parameter values are as shown in Table 1.

At time *t* = 0, all consumers start out with the same harvesting rate and dispersal radius. As the interplay of resource-consumer dynamics and strategy dynamics unfolds, consumers diversify into two distinct strategies (Fig 2). The first is a sedentary strategy, with consumers adopting a low harvesting rate (*r*_H_≈0.5) without dispersing (*σ*_D_≈0). The second is a mobile strategy, with consumers adopting a high harvesting rate (*r*_H_≈6) in combination with a non-zero dispersal radius (*σ*_D_≈1). At the population level, these two strategies keep coexisting over time, although individual consumers keep changing their strategies by imitating other consumers.

### Increasing consumption density leads to social inequality

**Fig 3:**
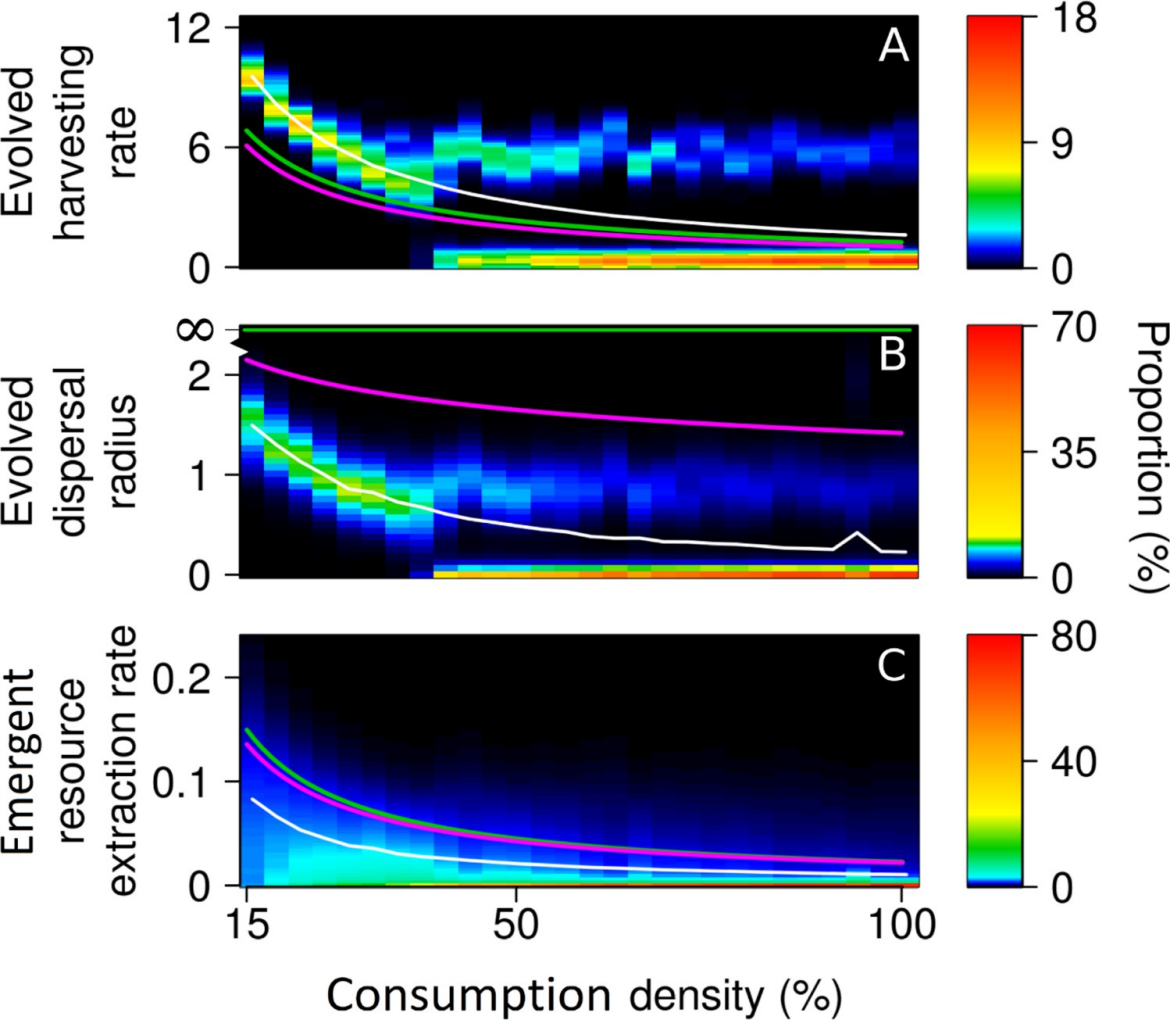
Increasing consumption density leads to social inequality. (A, B) When the density of consumers is low (expressed as the ratio of harvested area to total area), all of them evolve to harvest at high rates and disperse far, without overexploiting the resource. As consumption density increases, such a resource-consumption strategy becomes unsustainable, leading to a diversification of strategies into frugal sedentary consumers with low harvesting rates and near-zero dispersal radii, and overexploitative mobile consumers with high harvesting rates and large dispersal radii. (C) The resultant per capita resource extraction rates (expressed as fractions of the system’s carrying capacity extracted per unit time) decrease with consumption density. Population averages are indicated by white lines, yield-maximizing resource-consumption strategies and resultant resource extraction rates by green lines, and profit-maximizing resource-consumption strategies and resultant resource extraction rates by magenta lines. The green and magenta lines are exponential fits to numerically estimated strategy values, as in S2F Fig. The average harvesting rates exceed the corresponding yield-maximizing and profit-maximizing harvesting rates, implying overexploitation of the resource and suboptimal per capita resource extraction rates. Parameter values are as shown in Table 1.

When consumption density is low, all consumers can adopt an ‘affluent’ harvest-intensively-and-disperse-far strategy, enabling everyone to harvest at high rates without globally depleting the resource. As consumption density increases, harvesting rate and dispersal radius evolve to lower values (Fig 3). As consumption density increases further, the harvest-intensively-and-disperse-far strategy is no longer sustainable, because it begins to deplete the resource globally. Under these conditions, one might naively expect that all consumers would lower their harvesting rates to adjust to the changed circumstances. Instead, however, some consumers harvest even more intensively to sustain their costly dispersal, while other consumers harvest much less intensively and adopt a harvest-frugally-and-do-not-disperse strategy, thus accepting slower resource extraction along with reduced dispersal costs. The proportion of such sedentary consumers continues to increase when consumption density is increased further, until all consumers adopt a sedentary strategy.

As is typical in game-theoretic models, the average evolved harvesting rates (white curves in Fig 3) are higher, at all consumer densities, than the corresponding baseline rates, i.e., the yield-maximizing rates (green curves in Fig 3; see S2 Appendix for their formal definition) and the profit-maximizing rates (magenta curves in Fig 3; see S2 Appendix for their formal definition). Consequently, the average resource extraction rates are below their optimal values. Thus, when left to themselves, consumers overexploit the resource to some degree. From this observation, the following question arises: Under what circumstances do consumers adopt a yield-maximizing strategy?

### Mobile consumers achieve maximum yield at the ‘edge of inequality,’ unless dispersal is very cheap

**Fig 4:**
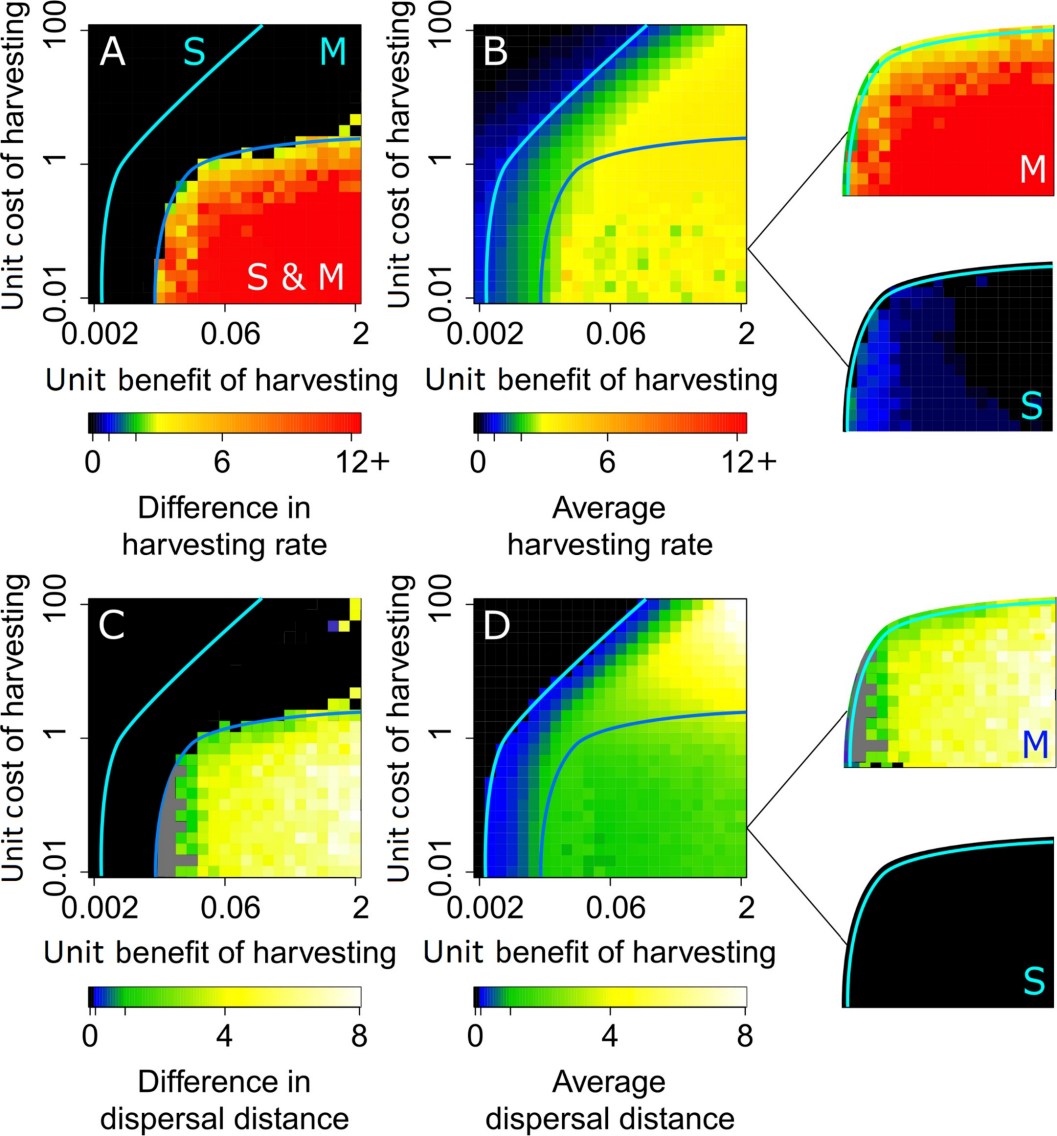
Strategy regimes for different unit benefits and costs of harvesting. Effects of the unit benefit *b*_H_ and cost *c*_H_ of harvesting on the (A) difference between harvesting rates of sedentary and mobile consumers, (B) population average of harvesting rates, (C) difference between dispersal distances of sedentary and mobile consumers, and (D) population average of dispersal distances. In panels A and B, the sedentary and mobile yield-maximizing harvesting rates (S2 Appendix) are indicated, respectively, by the unlabeled tick marks in the blue and green ranges of the color bars. In panel C, grey areas indicate where a difference in dispersal distances could not be reliably detect, due to insufficient numerical resolution. We find three strategy regimes, delimited by cyan and blue lines: in the region labeled ‘S,’ all consumers are prudent and sedentary, in the region labeled ‘M,’ all consumers are overexploitative and mobile, and in the region labeled ‘S & M,’ frugal sedentary consumers are coexisting with overexploitative mobile consumers. For the latter regime, outsets in panels B and D show average values separately for sedentary and mobile consumers. Parameter values are as shown in Table 1.

**Fig 5:**
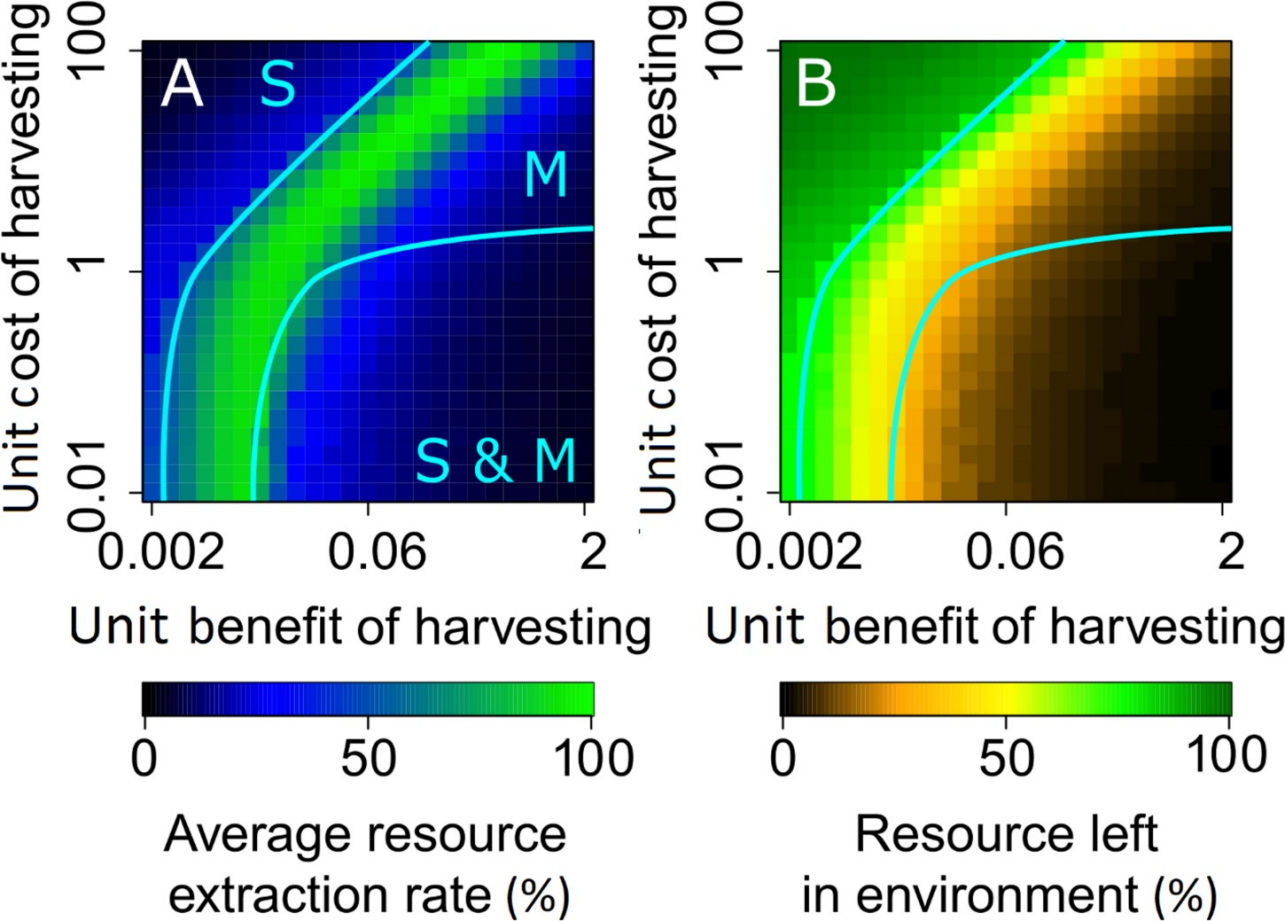
Maximum sustainable resource extraction occurs for mobile consumers at the ‘edge of inequality’. (A) Average per capita resource extraction rate (expressed as a fraction of the yield-maximizing extraction rate) and (B) amount of resource left in the environment (expressed as a fraction of the system’s carrying capacity), depending on the unit benefit *b*_H_ and cost *c*_H_ of harvesting. The sedentary regime, labeled ‘S,’ is equitable, but inefficient: the resource extraction is far less than optimal, despite the ample amounts of resource left in the environment. The mobile regime, labeled ‘M,’ is both equitable and efficient: the total resource extraction rate resulting from the evolved resource-consumption strategies reaches its maximum in this regime. However, for high unit benefits and costs of harvesting (or equivalently, for low unit costs of dispersal), the tragedy of the commons can occur also in this regime. The coexistence regime, labeled ‘S & M,’ is neither equitable nor efficient: the resource extraction rate is far less than optimal, and overexploitation by mobile consumers results in the tragedy of the commons. Parameter values are as in Fig 4.

When the unit benefit of harvesting is low relative to the unit cost of dispersal, the population’s strategy distribution is unimodal, with all consumers adopting a low harvesting rate (Fig 4B) and a near-zero dispersal radius (Fig 4D). This ‘sedentary regime’ applies in the region labeled ‘S’ in Fig 4. In this regime, resource harvesting is inefficient: the harvesting rate is low (Fig 5A) despite an abundant supply of the resource being available in the environment (Fig 5B). This is because dispersal costs that are high relative to the benefits of harvesting force consumers to adopt a sedentary lifestyle that prevents them from dispersing to less occupied resource-rich areas. Since each consumer harvests the resource only in his/her neighborhood without moving, consumers experience very little resource competition: thus, their resource extraction rates are similar and the resource distribution is fair.

As the unit benefit of harvesting is increased relative to the unit cost of dispersal, dispersal becomes viable. The population’s strategy distribution still remains unimodal, but all consumers now adapt a mobile (harvest-intensively-and-disperse-far) strategy. This ‘mobile regime’ applies in the region labeled ‘M’ in Fig 4. The harvesting rate and resource extraction rate both increase, until maximum yield is reached (green regions in Fig 4B and Fig 5A). At this resource extraction rate, consumers locally deplete the resource, but the depletion is temporary and resource extraction is globally sustainable. The resource distribution is fair, as all consumers harvest the resource at similar rates. Therefore, resource extraction is efficient as well as fair. However, when the unit benefit and cost of harvesting are both very high (or, equivalently, the unit cost of dispersal is very low), the temptation to harvest the resource rapidly is strong, as well as feasible, since dispersal is very cheap. Consequently, all consumers adopt a high dispersal radius and overexploit the resource: even though the population’s strategy distribution remains unimodal, resource extraction drops below optimal values as a consequence of the tragedy of the commons (top-right corners in the panels of Figs 4 and 5).

When the unit cost of harvesting is significant, but not too high, increasing the unit benefit of harvesting leads to a diversification of strategies, adopted by sedentary and mobile consumers. This ‘coexistence regime’ applies in the region labeled ‘S & M’ in Fig 4. Initially, mobile consumers tend to harvest at high rates. But as the amount of resource in the environment is diminished because of overexploitation by these consumers, the sedentary strategy becomes increasingly viable. This is because the benefits forgone by a reduction in harvesting rate are balanced by avoiding the costs of dispersal. As some consumers become sedentary, the density of mobile consumers decreases, allowing them to sustain higher harvesting rates. Therefore, frugal sedentary consumers (whom we might call cooperative) and overexploitative mobile consumers (whom we might call cheating) coexist in the population (Fig 4A and 4C). The coexistence regime is characterized by a social divide, in which the resource distribution is unfair. Low costs of harvesting allow the cheating consumers greatly to increase their harvesting rates (outsets in Fig 4B). Consequently, a small fraction of cheating consumers can thrive at the expense of a large fraction of cooperative consumers (S3 Fig).

For a low efficiency of resource harvesting, measured by the ratio *b*_H_/*c*_H_ of the unit benefit and cost of harvesting, sedentary consumers adopt the profit-maximizing harvesting rate (blue region in the outset labeled ‘S’ in Fig 4B): we thus call them prudent sedentary consumers. However, as this efficiency increases, mobile consumers become increasingly overexploitative (red region in the outset labeled ‘M’ in Fig 4B), destroying the resource even in the neighborhoods of the sedentary consumers: this drives the latter’s resource extraction rates to zero (black region in the outset labeled ‘S’ in Fig 4B), forcing them into being frugal sedentary consumers. In this regime, the resource distribution between sedentary and mobile strategies is highly inequitable.

### Rapid consumer adaptation and localized information aggravate social inequality

**Fig 6:**
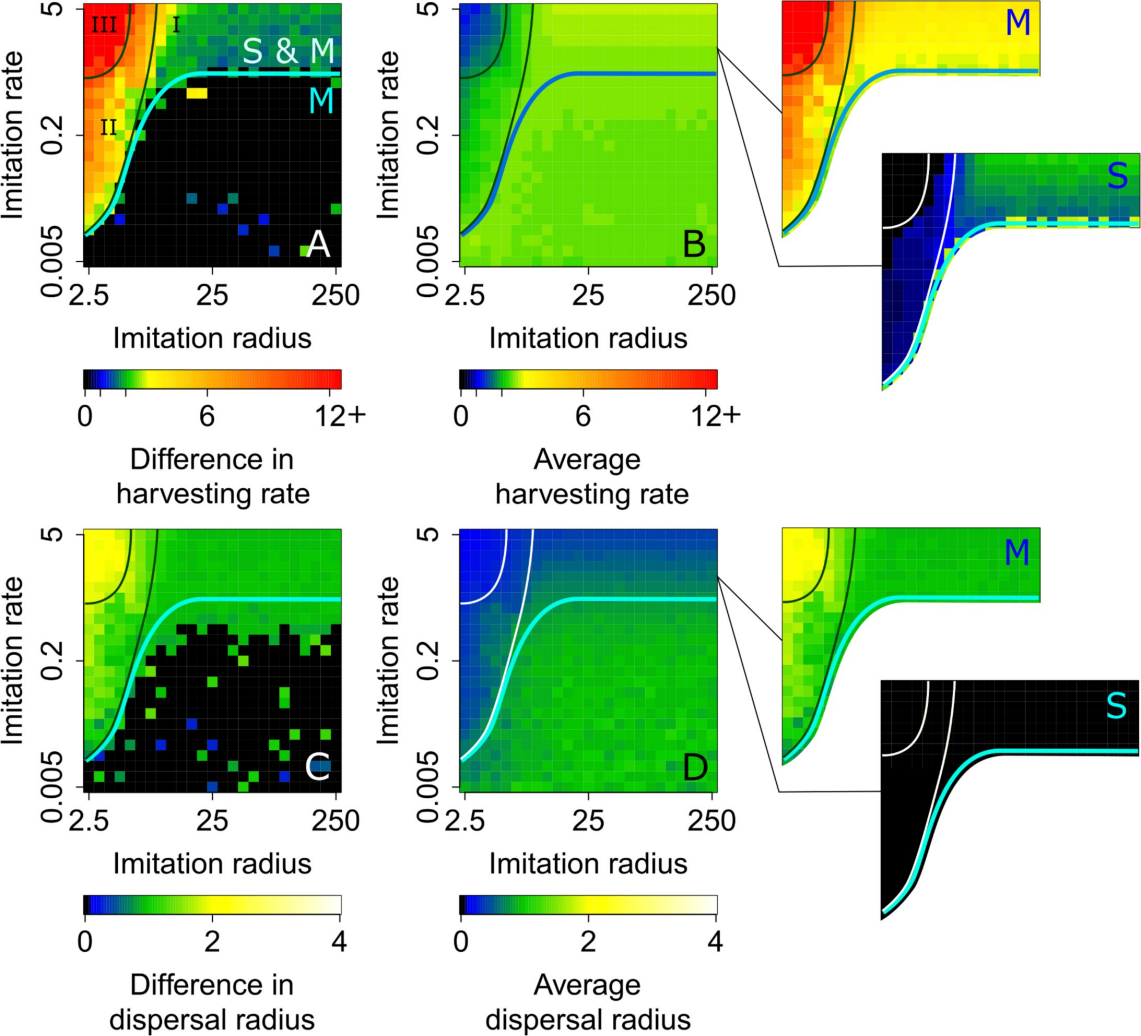
Strategy regimes for different spatio-temporal scales of imitation. Effects of the imitation radius *σ*_I_ and imitation rate *r*_I_ on the (A) difference between harvesting rates of sedentary and mobile consumers, (B) population average of harvesting rates, (C) difference between dispersal distances of sedentary and mobile consumers, and (D) population average of dispersal distances. In panels A and B, the sedentary and mobile yield-maximizing harvesting rates (S2 Appendix) are indicated, respectively, by the unlabeled tick marks in the blue and green ranges of the color bars. We find four strategy regimes, delimited by cyan, blue, black, and white lines. In the region labeled ‘M,’ characterized by relatively low imitation rates and not too small imitation radii, the population’s strategy distribution is unimodal, featuring an efficient mobile resource-consumption strategy. Increasing the imitation rate is taking the population into the coexistence region, labeled ‘S & M,’ in which strategies diversify, and sedentary and mobile consumers coexist. For the latter regime, outsets in panels B and D show average values separately for sedentary and mobile consumers. We further divide the region labeled ‘S & M’ into three regions. In the region labeled ‘I,’ characterized by global information, both sedentary and mobile consumers have high harvesting rates, resulting in the tragedy of the commons. Decreasing the imitation radius is taking the population into the region labeled ‘II,’ in which the mobile cheating consumers are becoming less common but more overexploitative, while sedentary cooperative consumers are harvesting at rates close to the sedentary profit-maximizing rate. Decreasing the imitation radius even further is taking the population into the region labeled ‘III,’ in which the mobile cheating consumers are becoming even less common but extremely overexploitative. S5 Fig provides additional information on the regions labeled ‘I’ to ‘III.’ Parameter values are as shown in Table 1, except for *b*_H_ = 0.02 and *c*_H_ = 0.8.

The imitation of resource-consumption strategies by consumers depends on their knowledge of the strategies of other consumers. The farther information on other consumers’ strategies travels, the larger is the pool of potential strategies each consumer can compare with its own and imitate. Up to now, we have assumed that all consumers have knowledge about strategies in the entire population (corresponding to an infinite imitation radius, *σ*_I_→∞). We now relax this assumption. Fig 6 shows how the evolved strategies depend on imitation radius and imitation rate, for payoff parameters from the mobile regime; S4 Fig provides an analogous analysis for payoff parameters from the coexistence regime.

**Fig 7:**
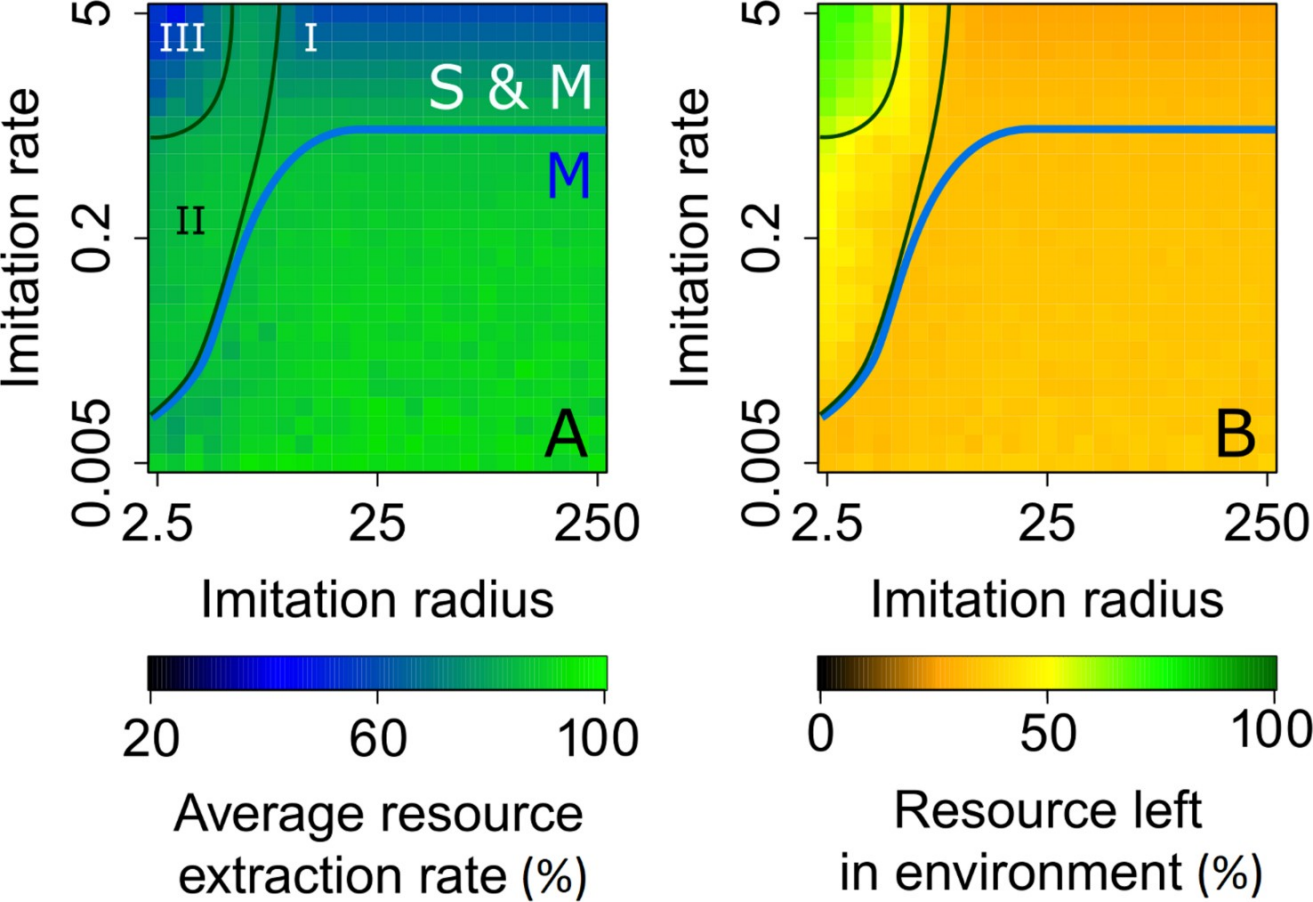
Consumer impatience and myopia aggravate social inequality. Effects of the imitation radius *σ*_I_ and imitation rate *r*_I_ on the (A) average per capita resource extraction rate (expressed as a fraction of the yield-maximizing extraction rate) and (B) amount of resource left in the environment (expressed as a fraction of the system’s carrying capacity). The average per capita resource extraction rate decreases as the imitation rate is increased. In the region labeled ‘III,’ this is because a small fraction of mobile cheating consumers is exploiting the resource at a very high harvesting rate, while sedentary cooperative consumers, which form the vast majority of the population, adopt a frugal harvesting rate and receive hardly any resource. In the region labeled ‘I,’ sedentary and mobile consumers are present in roughly similar proportions, both harvesting aggressively and overexploiting the resource. In the region labeled ‘II,’ a smaller, but still substantial, proportion of cheating consumers is enabled to coexist with the cooperative consumers. S5 Fig provides additional information on the regions labeled ‘I’ to ‘III.’ Parameter values are as in Fig 6.

For low to moderate imitation rates and not too small imitation radii (in the region labeled ‘M’ in Fig 6A), the population’s strategy distribution is unimodal (Fig 6A) and the resource extraction rate is high (Fig 7A), as characterizing the mobile regime. As the imitation rate increases, i.e., as consumers become more ‘impatient,’ they diversify into two subpopulations adopting sedentary or mobile strategies, respectively (in the region labeled ‘S & M’ in Fig 6A), as characterizing the coexistence regime. Fast imitation causes a strategy to change even before its consequences on the resource are reflected in the payoffs consumers receive. This can prevent sedentary consumers from realizing the long-term benefits of their low harvesting rates, which can lead to an unsustainable increase in their harvesting rates (green region in the outset labeled ‘S’ in Fig 6B). For large imitation radii (in the region labeled ‘I’ in Fig 6A), the differences between the harvesting rates of sedentary and mobile consumers are relatively small (S5A Fig). However, since the proportion of mobile consumers is relatively high (S5 Fig) and sedentary consumers also exploit at rates higher than the sedentary yield-maximizing rate (S5A Fig), the resource is overexploited (Fig 7B).

The mobile strategy becomes more rewarding when consumers are not only impatient, but also myopic (in the region labeled ‘III’ in Fig 6A). Under such circumstances, the cheating consumers become extremely overexploitative, with very high harvesting rates (red region in the outset labeled ‘M’ in Fig 6B). However, their proportion is low (S5 Fig). This implies a stark social divide, with a handful of cheating consumers driving the extraction rates of the majority of consumers close to zero. Resource harvesting is acutely inefficient, with much of the resource being left unharvested (green region in Fig 7B).

For intermediate spatio-temporal scales of imitation (in the region labeled ‘II’ in Fig 6A), cheating consumers are less aggressive, but more abundant. The harvesting rates of cooperative consumers are close to their sedentary profit-maximizing values. This leads to greater resource extraction (Fig 7A), almost entirely by the cheating consumers (Figs 6B and S5A). Consequently, this region is most favorable for cheating consumers.

### Results are robust to altering model parameters and model structure

To investigate the robustness of our results under more realistic assumptions for intelligent consumers, we allow consumers to explore random strategies, in addition to imitating the strategies of role models, by adopting harvesting rates and dispersal radii randomly drawn, at the chosen strategy-exploration rate, from salient uniform distributions. S6 Fig shows that, for sufficiently low strategy-exploration rates, the population of consumers evolves to the same strategy combination as in Fig 2, which indicates that the strategy dynamics has no hidden attractors that could be attained only through strategy exploration.

After some social learning, the resource-consumption strategies in the consumer population typically exhibit a slight spread around one or two modes (Figs 2–5). This spread is largely determined by the implementation errors *μ*_H_ and *μ*_D_ of strategy imitation and by the duration *T* of payoff averaging, whose values we have chosen in accordance with the need for computational efficiency. It is therefore instructive to demonstrate that the evolved mean strategies are robust under altering these parameters: as expected, using smaller imitation errors and longer averaging durations results in more sharply peaked strategy distributions, without noticeably affecting the modes’ mean strategies (S7 Fig).

We can relax the assumption that consumers imitate the strategies of other consumers if and only if those receive a higher payoff, by consider a smooth imitation-response function *S*_I_(Δ*V*) = 1/(1+exp(−*k*_I_Δ*V*)), instead of the step function (*k*_I_→∞) used for our main results. Having chosen another consumer as a potential role model, the probability of imitating him/her is now *S*_I_(Δ*V*), where Δ*V* is the difference between the chosen consumer’s payoff and the focal consumer’s payoff. An increased smoothness of the imitation-response function, resulting for lower values of *k*_I_, can describe an increased reluctance to adopt a higher-payoff strategy in conjunction with an increased willingness to try out a lower-payoff strategy. It can also describe increases in errors of perception and/or implementation, which arise, respectively, when consumers cannot accurately assess payoffs or cannot accurately implement the decision whether or not to imitate a role model. S8 Fig shows that our results are robust to a reasonable degree of imitation-response smoothness. However, too much smoothness leads to the widespread adoption of significantly suboptimal strategies, such as a high harvesting rate without dispersal.

We can relax the assumption that consumers disperse if and only if the resource density at their locations drop below the threshold *R*_T_, by considering a smooth dispersal-response function *S*_D_(Δ*R*) = 1/(1+exp(*k*_D_Δ*R*)), instead of the step function (*k*_D_→∞) used for our main results. This function determines a consumer’s rate of dispersing away from his/her location in dependence on the difference Δ*R* = *R*−*R*_T_ between the local resource density *R* and the threshold *R*_T_. Possible causes for smoothness in the dispersal response are analogous to those impinging on the imitation response. S9 Fig shows that our results are robust to considerable changes in the dispersal-response smoothness.

We can relax the assumption that there is no intrinsic spatial heterogeneity in the spatial distribution of resources, by describing spatially heterogeneous resource growth rates (S10 Fig) using a synthetic turbulence model [52]. When dispersal is not costly or imitation is slow or global, our results are robust to spatial heterogeneity, but when dispersal is costly and imitation is fast and local, spatial heterogeneity may lead to strategy diversification and inefficient resource extraction (S11 Fig). This happens only when the imitation radius is smaller than the distance over which the resource growth rate varies significantly, as quantified by the spatial correlation length of the resource growth rate. We can understand this result by appreciating that small imitation radii together with high imitation rates enable the local adaptation of resource-consumption strategies: when the imitation radius is smaller than the correlation length, this allows for the evolution of consumers specializing on low and high resource growth rates, respectively. In contrast, when the correlation length is smaller than the imitation radius, or when imitation is slow, consumers experience a diversity of resource growth rates before imitating. Thus, the effects of spatial heterogeneity are averaged out and the evolved strategies are similar to those arising in an intrinsically homogeneous space.

We also investigate the impacts on evolved strategies of altering our model so as to describe more sophisticated consumers. In particular, we consider two additional scenarios. First, sedentary consumers may have the capacity to increase the resource growth rate or to generate additional resource (such as when agriculture utilizes external supplies of energy and nutrients). Second, mobile and sedentary consumers may be slightly or fully specialized on harvesting different resources (such as when farmers and hunter-gatherers differ in their diets). In other words, sedentary consumers might be able to achieve a greater resource extraction rate than mobile consumers, and mobile consumers might not be able equally to exploit the resource used by sedentary consumers. To explore these scenarios in a simple way, we assume that sedentary consumers benefit from a fixed additional supply *R*_G_ of resource per unit time. S12 Fig shows that such additional resource acquisition by sedentary consumers does not qualitatively alter the evolutionary outcomes. Quantitatively, mobile consumers evolve higher extraction rates to match the additional resource acquisition of sedentary consumers. Further, as the sedentary strategy becomes more profitable through additional resource acquisition, the mobile regime shrinks or disappears: when the rate described by *R*_G_ is much greater than the yield-maximizing harvesting rate of mobile consumers, all consumers become sedentary; otherwise, sedentary and mobile consumers coexist.

## Discussion

**Fig 8:**
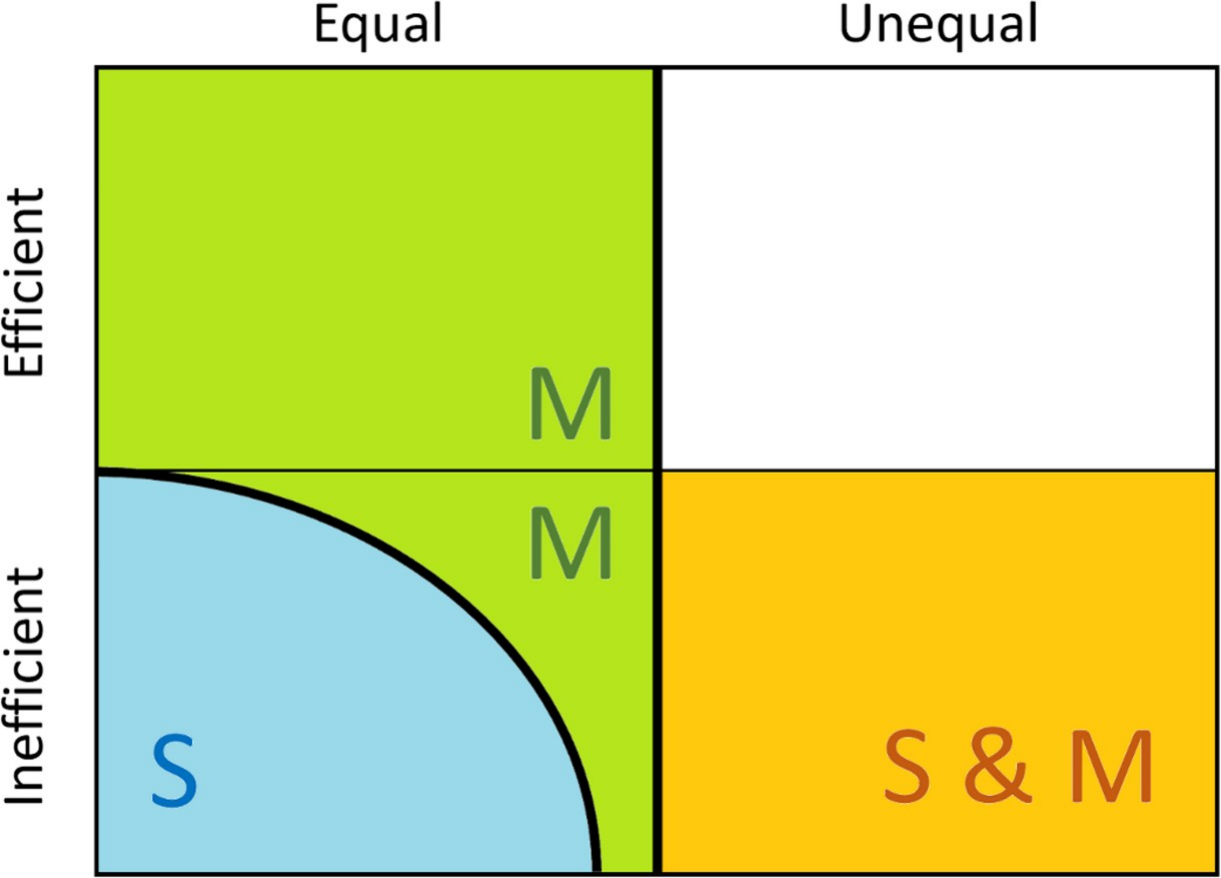
Overview of harvesting and dispersal regimes and their ranges in equality-efficiency space. The sedentary regime (labelled ‘S’) is equitable but inefficient, the mobile regime (labelled ‘M’) is equitable and efficient at high dispersal costs but inefficient at low dispersal costs, while the coexistence regime (labelled ‘S & M’) is neither equitable nor efficient.

We have shown that differences in resource productivity lead to different emerging harvesting regimes, including a sedentary regime, which is equitable but inefficient, a mobile regime, which is equitable and efficient at high dispersal costs but becomes inefficient as dispersal costs are reduced, and a coexistence regime, which is neither equitable nor efficient (Fig 8). The inefficiency in the sedentary regime results from consumers individually lacking the ability to harvest resources efficiently, whereas the inefficiency in the mobile regime and coexistence regime results from consumers collectively harming each other’s harvesting efficiency through competition, i.e., through the tragedy of the commons. Therefore, mobility as such is desirable, as it leads to equitable and efficient resource use, but excessive mobility is harmful, as it leads to unsustainable resource extraction. We have further shown that when consumers are impatient and myopic, the social inequality between cheating consumers and cooperative consumers is aggravated. Thus, high resource productivity, in conjunction with consumer impatience and myopia, are the key features that cause social divides, inefficient resource extraction, and global overexploitation of the resources.

### Comparison with previous studies

Spatial public goods games have frequently been used to study the evolution of cooperation. Often, models of spatial public goods rely on additional mechanisms to stabilize cooperation, such as volunteering [35], rewarding cooperators or punishing defectors [28, 53, 54], or conditional strategies [55]. Other models rely on spatial structure, intrinsic or emergent, that supports the clustering of cooperators [36, 56, 57]. In all these studies, cooperation has been defined through its effects on the game-theoretical payoffs, and whether an act is cooperative or defective is independent of the prevailing socio-ecological conditions. By contrast, cooperation and defection in our model can only be defined in relation to the resource: a resource-consumption strategy is cooperative if and only if it ensures that, when adopted by all consumers in the population, the resource extraction rate is less than that giving maximum sustainable yield.

When movement is costly, consumers of a spatial resource may face a ‘milker-killer dilemma’ [58, 59], in which each consumer has a choice to adopt a ‘milker’ strategy (like our sedentary consumers) or a ‘killer’ strategy (like our mobile consumers). Several earlier studies have tried to identify the conditions under which either milker or killer strategies are favored [58, 60, 61]. But none of these studies had found coexistence of milkers and killers. A recent study [46] reported the coexistence of sessile cooperators (like milkers) and mobile defectors (like killers), by allowing the coevolution of cooperation and unconditional dispersal, and concluded that such coexistence is favored when dispersal costs are low. In broad agreement with the results of that study, we have demonstrated the emergence and coexistence of frugal sedentary consumers and mobile overexploitative consumers. However, in contrast to that study, we have found that in our system, coexistence is favored when dispersal costs are high, whereas consumers all tend to be mobile and overexploitative when dispersal costs are low. This is because dispersal opens up the possibility of overexploiting local resources and of finding new resource-dense areas to exploit. Furthermore, interactions in our model are entirely mediated by the resource dynamics. Specifically, if the resources were inexhaustible, consumers who harvest aggressively and do not disperse would outcompete those with other strategies. Therefore, the exhaustible and renewable nature of resources in our model is essential for the evolution of dispersal.

Another recent study [45] used an infinite island model to study the coevolution of cooperation (quantified in terms of investments to public goods) and dispersal (quantified in terms of the rate of leaving the current patch). That study found that both dispersal and cooperation are favored if catastrophes wipe out local populations at an intermediate rate. This is because catastrophes reduce local population densities and increase relatedness among the remaining consumers. It would be interesting to examine the effects of catastrophes in our model. On the one hand, catastrophes may prevent strategy diversification because a decrease in consumption density brought about by a catastrophe may allow all consumers to sustain high harvesting rates. On the other hand, catastrophes may facilitate coexistence due to the well-known competition-colonization tradeoff.

### Directions for future research

Our model makes deliberately simple assumptions to capture the essential processes governing resource consumption and strategy evolution. For example, we have assumed that all consumers have the capacity to adopt any strategy they wish, and without delay. Thus, a sedentary consumer with a very low harvesting rate can quickly switch to a mobile strategy that is much more expensive in terms of the costs of harvesting and dispersal. In real situations, however, resource-poor consumers may not be able to afford a strategy switch, forcing them into a low-consumption strategy permanently, or they might be able to afford such a switch only after accumulating sufficient payoff, forcing them into implementing the intended switch with a delay. These effects could be investigated by extending our model to incorporate costly strategy switches.

Another direction for future research is to allow the imitation rate to evolve and to investigate whether consumers then adopt a fast or slow imitation rate. We speculate that without a cost of imitation, consumers will evolve to be increasingly impatient, as they (and especially those among them that happen to have lower payoffs) will benefit from increased opportunities to imitate more successful strategies. Nonetheless, multiple strategies may still exist, even when consumers rapidly switch between them, as we have already observed in the present study.

Although we have investigated the dependence of evolved resource-consumption strategies on consumer density, we have kept the number of consumers constant within individual model runs. However, it is well known that increased resource consumption resulting from higher efficiency and better technology often leads to increases in population size, and thus, consumer density. This, in turn, may necessitate and enable further increases in harvesting efficiency. It is possible to incorporate such additional feedbacks into our model, and exploring the fate of a society under such circumstances therefore constitutes a particularly interesting avenue for further research.

### Conclusions

In conclusion, we have explored how the dynamics of a renewable resource influence the harvesting and dispersal strategies of consumers. We have found that high consumption density, high efficiency of resource harvesting, low costs of dispersal, rapid strategy adaptation among consumers, and localized information about the strategies of other consumers, are features that lead to spontaneous diversification of consumers into frugal sedentary consumers and overexploitative mobile ones. This inequitable social divide is always accompanied by an overexploitation of the resource.

In modern societies, developments involving technological progress have led to an increased efficiency of resource harvesting and to a reduced cost of dispersal. This has led, in turn, to an increase in population size. Furthermore, the pace of modern life has quickened, which in our model can be accounted for by an increased pace of strategy adaptation. While these are positive developments as such, our analyses suggest that all four of them–individually, and especially together–pose a threat to social equality and the sustainability of resource consumption. Our results also suggest a specific way of mitigating these threats: an increased spatial scale of information availability, which is another characteristic feature of modern societies, can safeguard social equality and promote sustainable resource consumption. We hope that further work can build on the findings presented here and shed light on the foundations of human and animal behavior and their changes in response to changing socio-ecological conditions.

## Supporting information

S1 Table Glossary. (PDF)

S1 Appendix Choice of units and essential parameters. (PDF)

S2 Appendix Optimal strategies. (PDF)

S1 Video Consumer-resource dynamics. Schematic video showing consumers (red squares) harvesting a renewable resource according to an exploitation kernel as described by Eq 2. Resource density is represented by greyscale, with black corresponding to zero resource density and white corresponding to the carrying-capacity density. Consumers disperse when the local resource density falls below the resource-density threshold for dispersal. This schematic video illustrates the mobile regime, in which all consumers are similarly mobile.
(MP4)

S1 Fig Evolved average harvesting rates are always lower than the corresponding profit-maximizing harvesting rates. Effects of the unit benefit *b*_H_ and cost *c*_H_ of harvesting on the profit-maximizing (A) harvesting rates and (C) dispersal radii, and on the resultant (B) per capita resource extraction rates and (D) dispersal distances. In panel A, the sedentary and mobile yield-maximizing harvesting rates (S2 Appendix) are indicated, respectively, by the unlabeled tick marks in the blue and green ranges of the color bars. Comparing Figs 4B and 5A with panels A and B, respectively, shows that the evolved average harvesting rates and resource extraction rates are always lower than, respectively, the profit-maximizing harvesting rates and resource extraction rates, especially in the coexistence region, labeled ‘S & M’. Parameter values are as in Figs 4 and 5, while imitation parameters are not relevant to this figure.
(TIF)

S2 Fig Maximum yield is obtained through global dispersal and harvesting rates exceeding the resource’s intrinsic growth rate. (A, B) Yield-maximizing resource-consumption strategies are found by computing and comparing the per capita resource extraction rates (expressed as fractions of the system’s carrying capacity extracted per unit time) of monomorphic populations with different harvesting rates and dispersal radii, here shown for a consumption density of 50.8% (expressed as the ratio of harvested area to total area). (C) The per capita resource extraction rate is maximized at an intermediate harvesting rate, called the yield-maximizing harvesting rate. (D) The resource-extraction rate increases monotonically with the dispersal radius, i.e., the yield-maximizing dispersal radius is infinite. (E-F) The yield-maximizing harvesting rate and the resultant maximum per capita resource extraction rate both decrease with consumption density. The maximum resource extraction rate is inversely proportional to the consumption density, with a power-law exponent close to –1. This indicates minimal kernel overlap among mobile consumers, i.e., mobile consumers evade through movement configurations in which their exploitation kernel overlaps with that of another consumer. Parameter values are as shown in Table 1, while imitation parameters are not relevant to this figure.
(TIF)

S3 Fig As the efficiency of resource harvesting is increased, the evolved resource -consumption strategies change from sedentary to mobile to a coexistence of sedentary and mobile. Effects of the unit benefit *b*_H_ of harvesting on the evolved population distributions of (A) harvesting rates, (B) dispersal radii, (C) dispersal distances, and (D) per capita resource extraction rates. The blue curve in panel D shows the average per capita resource extraction rate. We can clearly see the three strategy regimes: sedentary (labelled ‘S’), mobile (labelled ‘M’), and coexistence of sedentary and mobile (labelled ‘S & M’). Parameter values are as in the row with *c*_H_ = 0.68 in Fig 4 (Fig 4C and 4D show only dispersal distances and not also dispersal radii because the latter tend to drift in the region labeled ‘S,’ where the former are very small, as sustainable harvesting ensures that the resource densities at the locations of consumers very rarely fall below the dispersal threshold).
(TIF)

S4 Fig Impatient myopic imitation aggravates the inequality between sedentary and mobile consumers. Effects of the imitation radius *σ*_I_ and imitation rate *r*_I_ on the (A) difference between harvesting rates of sedentary and mobile consumers, (B) population average of harvesting rates, (C) difference between dispersal radii of sedentary and mobile consumers, (D) population average of dispersal radii, (E) average per capita resource extraction rate (expressed as a fraction of the yield-maximizing extraction rate), and (F) amount of resource left in the environment (expressed as a fraction of the system’s carrying capacity). While Figs 6 and 7 show how fast local imitation can lead to strategy diversification that does not occur for slow global imitation, here we show that this finding extends to parameter combinations in the coexistence region, where fast local imitation aggravates the aforementioned social inequality. For very low imitation rates (black regions in panels A and C), strategy diversification does not occur within the limited duration of the model runs considered here. Parameter values are as shown in Table 1.
(TIF)

S5 Fig Mobile consumers are most successful at intermediate imitation radii. Effects of the imitation radius on the evolved population distributions of (A) harvesting rates, (B) dispersal radii, (C) dispersal distances, and (D) per capita resource extraction rates, for a large imitation rate corresponding to the top rows of panels in Fig 6. The blue curves in all panels show averages of the population distributions, while the red and green curves in panels A to C show averages of the population distributions separately for the two modes. We see that the per capita resource extraction rate is highest in the region labelled ‘II,’ in which the imitation radius is intermediate. For smaller imitation radii in the region labelled ‘III,’ the mobile consumers are very few but highly overexploitative, and therefore still drive the average resource extraction rate of the sedentary consumers to close to zero. For larger imitation radii in the region labelled ‘I,’ the sedentary consumers are also overexploitative, and the population’s average per capita resource extraction rate drops accordingly. Parameter values are as shown in Table 1, except for *r*_I_ = 5.
(TIF)

S6 Fig Occasional strategy exploration does not alter evolutionary outcomes. Time series of population distributions of harvesting rates for five different strategy-exploration rates shown in the rightmost column (expressed as fractions of the imitation rate), shown alongside the time-averaged population distributions (averaged over the last 1,000 units to exclude the initial transient), with the frequencies in the separate bins adding up to the number *N* of consumers. Upon an exploration event, consumers change their harvesting rates and dispersal radii to values randomly drawn from uniform distributions over the intervals [0, 12.5] and [0, 2.5], respectively. For strategy-exploration rates of up to about 2% of the imitation rate, the evolutionary outcome remains qualitatively unchanged, with the two modes corresponding to frugal sedentary consumers (labeled ‘S’) and overexploitative mobile consumers (labeled ‘M’), respectively. Higher strategy-exploration rates widen these modes to an extent that the separation between them is lost. Parameter values are as shown in Table 1, except for *c*_H_ = 0.68.
(TIF)

S7 Fig Results are robust to altering the mutation-step size and payoff-averaging duration. The left column shows time series of the population distributions and time-averaged distributions of harvesting rates and dispersal radii for the default parameter values, while the right column shows the same information for smaller imitation errors and longer payoff-averaging durations. These time series are shown alongside the time-averaged population distributions (averaged over the last one third of the total simulated duration to exclude the initial transient, with the frequencies in the separate bins adding up to the number *N* of consumers). The two rows show results for two different imitation rates, *r*_I_ = 0.5 in the upper row and *r*_I_ = 0.15 in the lower row. As expected, the evolutionary dynamics in the right column are slower and the resultant modes are narrower than in the left column. Nevertheless, the evolutionary dynamics in the two columns are analogous, exhibiting the same pattern of diversification into sedentary and mobile resource-consumption strategies and leading to roughly the same average harvesting rates and dispersal radii in the two modes. Parameter values are as shown in Table 1, except for *T* = 1, *μ*_H_ = 0.0125, and *μ*_D_ = 0.00125 in the right column.
(TIF)

S8 Fig Results are robust to perception errors in the imitation response, unless these become too large. Effects of the smoothness of the imitation-response function on the evolved population distributions of (A) harvesting rates, (B) dispersal radii, (C) dispersal distances, and (D) per capita resource extraction rates. The smoothness is measured as *w*_I_/Δ*V*_95_, where the width *w*_I_ is the range of payoff differences over which the imitation-response function changes from 5% to 95%, and Δ*V*_95_ is the 5% to 95% quantile range of the payoff differences experienced by consumers. Our results are robust until the aforementioned width exceeds about a tenth of the experienced payoff differences. Higher smoothness promotes unsustainable resource-consumption strategies and causes overexploitation of the resource. Parameter values are as shown in Table 1.
(TIF)

S9 Fig Results are robust to perception errors in the dispersal response, even when these become very large. Effects of the smoothness of the dispersal-response function on the evolved population distributions of (A) harvesting rates, (B) dispersal radii, (C) dispersal distances, and (D) per capita resource extraction rates. The smoothness is measured as *w*_D_/Δ*R*_95_, where the width *w*_D_ is the range of resource densities over which the dispersal-response function changes from 5% to 95%, and Δ*R*_95_ is the 5% to 95% quantile range of the resource densities experienced by consumers. Our results are robust over several orders of magnitude of the dispersal-response smoothness. Parameter values are as shown in Table 1.
(TIF)

S10 Fig Results are robust to spatially heterogeneous resource growth. (A-C) Heterogeneous spatial distributions of the intrinsic resource growth rate with different spatial correlation lengths *L*_c_. The shown spatial distributions are generated using a synthetic turbulence model [52], the code for which can be found at https://github.com/jaideep777/Consumer-resource-system/blob/master/src/turbulence.cu.
(TIF)

S11 Fig Under fast local imitation, spatial heterogeneity causes strategy diversification, aggravating inefficient and inequitable resource harvesting. Effects of the imitation radius *σ*_I_ and correlation length *L*_c_ of heterogeneous spatial distributions of the intrinsic resource growth rate on the (A) difference between harvesting rates of sedentary and mobile consumers, (B) population average of harvesting rates, (C) difference between dispersal radii of sedentary and mobile consumers, (D) population average of dispersal radii, (E) average per capita resource extraction rate (expressed as a fraction of the yield-maximizing extraction rate), and (F) amount of resource left in the environment (expressed as a fraction of the system’s carrying capacity). For fast imitation, spatial heterogeneity causes strategy diversification roughly when the correlation length *L*_c_ exceeds the imitation radius *σ*_I_. Parameter values are as shown in Table 1, except for *b*_H_ =0.14, *c*_H_ = 0.8, and *r*_I_ = 0.5.
(TIF)

S12 Fig Mobile consumers persist even when sedentary consumers benefit from additional resource supply. Effects of the unit benefit *b*_H_ of harvesting on the evolved population distributions of harvesting rates for four different values of *R*_G_, the fixed additional supply of resource per unit time available to consumers who do not disperse, shown in the rightmost column (expressed as fractions of the maximum resource extraction rate of sedentary consumers shown in Equation 11). (A) For *R*_G_ = 0, the three strategy regimes shown in S3 Fig apply: sedentary (labelled ‘S’), mobile (labelled ‘M’), and coexistence of sedentary and mobile (labelled ‘S & M’). (B, C) Cases with *R*_G_>0 describe scenarios in which sedentary resource consumption enables increased ecological efficiency (e.g., through agricultural activities) or decreased competition with mobile consumers (e.g., through diet specialization). To offset the additional resource supply of the sedentary consumers, the mobile consumers harvest at higher rates, which enables them to keep coexisting with the sedentary consumers. (D) Only when the additional resource supply of the sedentary consumers is much greater than what is otherwise available to them, sedentary consumers outcompete mobile consumers. The red lines indicate how the boundaries between the three strategy regimes shift as *R*_G_ is raised from 0. Parameter values are as in S3 Fig.
(TIF)

S13 Fig Effects of exploitation-kernel overlap are small for consumption densities of up to 50%. (A) Maximum resource extraction rates of randomly placed sedentary consumers as a function of their harvesting rate, for four different consumption densities (red, magenta, blue, and black circles with fitted cubic splines). The orange line indicates the numerically calculated yield-maximizing harvesting rates (horizontal coordinates along orange line) and maximum resource extraction rates (vertical coordinates along orange line). These are compared with the corresponding analytical values (horizontal and vertical grey lines, respectively), calculated assuming no overlap of exploitation kernels (S2 Appendix). (B) Approximation accuracy, defined as the percentage drop in the maximum resource extraction rate (orange line) and the yield-maximizing harvesting rate (brown line) relative to their respective analytical values, as a function of consumption density. At low consumption densities, the maximum resource extraction rate and the yield-maximizing harvesting rate are both close to their respective analytical values, indicating that the effects of kernel overlap (cyan line) are small. The approximation accuracy remains higher than 75% even at 50% consumption density and decreases to no less than 60% at 100% consumption density. The relative kernel overlap is defined as the average fraction of a consumer’s exploitation area that overlaps with that of other consumers. Accordingly, the relative drop in maximum resource extraction rate mirrors the relative kernel overlap.
(TIF)
