# Supplementary material for: Emergence of social inequality in the spatial harvesting of renewable public goods
Source: PLoS Comput Biol. 2020 Jan 8;16(1):e1007483. doi: 10.1371/journal.pcbi.1007483 (PMC6974303; doi:10.1371/journal.pcbi.1007483)
Supplement: S1 Table — (PDF) [file pcbi.1007483.s001.pdf]

Term	Explanation
Efficiency of resource harvesting	Ratio of the unit benefit and cost of harvesting, $b_{\mathrm{H}}/c_{\mathrm{H}}$.
Utility of resource harvesting	Difference between the benefit and cost rates of harvesting, $b_{\mathrm{H}}R_{\mathrm{H},i} - c_{\mathrm{H}}r_{\mathrm{H},i}^2$.
Yield-maximizing resource-consumption strategy	For mobile consumers, the combination of harvesting rate and dispersal radius that maximizes their per capita resource extraction rate, assuming that the population is monomorphic (it turns out that the yield-maximizing dispersal radius is infinite). For sessile consumers, the harvesting rate that maximizes their per capita resource extraction rate given that their dispersal radius is zero.
Profit-maximizing resource-consumption strategy	For mobile consumers, the combination of harvesting rate and dispersal radius that maximizes their per capita payoff, assuming that the population is monomorphic (it turns out that the yield-maximizing dispersal radius is infinite). For sessile consumers, the harvesting rate that maximizes their per capita payoff given that their dispersal radius is zero.
Optimal resource-consumption strategy	Yield-maximizing or profit-maximizing strategy, depending on the context.
Inefficient resource harvesting	Harvesting through which the resource extraction rate is less than the resource extraction rate at maximum per capita yield.
Equitably and fair resource harvesting	Harvesting through which all consumers harvest the resource at an equal rate.
Sedentary consumers	Consumers with near-zero dispersal radius.
Mobile consumers	Consumers with large dispersal radius.
Frugal consumers	Consumers whose harvesting rate is considerably smaller than the yield-maximizing harvesting rate.
Prudent consumers	Consumers whose harvesting rate is roughly equal to the yield-maximizing harvesting rate.
Cooperative consumers	Consumers whose harvesting rate is smaller than or equal to the yield-maximizing harvesting rate.
Cheating consumers	Consumers whose harvesting rate is larger than the yield-maximizing harvesting rate, which implies that they overexploit the resource.
Impatient consumers	Consumers who imitate at rates faster than the intrinsic growth rate of the resource.
Myopic consumers	Consumers who have knowledge only of the strategies of nearby consumers and therefore can imitate only such strategies.

Table S1. Overview of key terms.
