# Supplementary material for: Emergence of social inequality in the spatial harvesting of renewable public goods
Source: PLoS Comput Biol. 2020 Jan 8;16(1):e1007483. doi: 10.1371/journal.pcbi.1007483 (PMC6974303; doi:10.1371/journal.pcbi.1007483)
Supplement: S1 Appendix — (PDF) [file pcbi.1007483.s002.pdf]

Appendix S1: Choice of units and essential parameters

We choose the units for length, time, resource density, and payoff to eliminate extraneous parameters and thus establish a minimal parameter set that contains only essential parameters. Specifically, we use the harvesting radius as the length unit, $\sigma_{\mathrm{H}} = 1$, and use the reciprocal of the intrinsic resource growth rate as the time unit, $r = 1$. The resource density is measured as a fraction of its carrying-capacity density, $K = 1$. Furthermore, we specify all payoffs relative to the unit cost of dispersal: therefore, the cost per unit dispersal length is assumed to be one payoff unit, $c_{\mathrm{D}} = 1$.

By measuring all quantities in these units, we obtain the parameter values shown in Table 1. The values of the system size L, the maximum dispersal rate $r_{\mathrm{D,\,max}}$, and the duration T over which payoffs are averaged, do not matter as long as they are chosen large enough (we chose L to be as large as possible given the state-of-the-art GPU-computing infrastructure). Likewise, the values of the implementation errors μ_{H} and μ_{D} for imitating harvesting rates and dispersal radii, respectively, do not matter as long as they are chosen small enough. Moreover, the value of the resource-density threshold R_{T} for dispersal does not matter as long as it is chosen not too close to either 0 or 1. Consequently, we are left with only five essential parameters. The first is the consumer density ρ, and the remaining four can be divided into two pairs, referring to payoff and imitation, in which b_{H} and c_{H} are the payoff parameters, and r_{I} and σ_{I} are the imitation parameters.

To facilitate interpretation, we express the resource extraction rate either as a fraction of the yield-maximizing extraction rate (Figures 2, 4-7, S1, S3-S5, S8-S9, S10-S11) or as a fraction of the system's carrying capacity extracted per unit time (Figures 3, S2). Likewise, we express time in units of the reciprocal of the default imitation rate (Appendix S1), the strategy-exploration rate as a fraction of the imitation rate, consumer density as the ratio of harvested area to total area, and the amount of resource left in the environment as a fraction of the system's carrying capacity.
