# Supplementary material for: Emergence of social inequality in the spatial harvesting of renewable public goods
Source: PLoS Comput Biol. 2020 Jan 8;16(1):e1007483. doi: 10.1371/journal.pcbi.1007483 (PMC6974303; doi:10.1371/journal.pcbi.1007483)
Supplement: S2 Appendix — (PDF) [file pcbi.1007483.s003.pdf]

Appendix S2: Optimal strategies

For comparing the evolved resource-consumption strategies to the strategies that maximize profit or yield for the same parameter values, we define the latter below.

S2.1 Yield-maximizing and profit-maximizing strategies of mobile consumers

A yield-maximizing strategy is a strategy that gives the highest possible per capita resource extraction rate of consumers in a monomorphic population (i.e., in a population in which all consumers adopt the same resource-consumption strategy). The yield-maximizing harvesting rate of mobile consumers is calculated numerically (Figure S2). For any fixed harvesting rate, the resource extraction rate increases with the dispersal radius (Figures S2A and S2D). We therefore define the yield-maximizing harvesting rate of mobile consumers as the rate that maximizes resource extraction at infinite dispersal radius. Note that the realized yield is often far lower when the cost of dispersal prevents consumers from dispersing far. Naturally, the yield-maximizing harvesting rate is inversely proportional to consumption density. Figure S2E shows yield-maximizing harvesting rates as a function of consumption density.

A profit-maximizing strategy is a strategy that gives the highest possible per capita payoff to consumers in a monomorphic population. The profit-maximizing harvesting rate of mobile consumers is also calculated numerically (Figure S1). Since both harvesting rate and dispersal radius are costly traits, the profit-maximizing harvesting rate and resource extraction rate are lower than their corresponding yield-maximizing values. Figure S1 shows profit-maximizing strategies as a function of the payoff parameters.

S2.2 Yield-maximizing and profit-maximizing strategies of sedentary consumers

If a consumer i is sedentary, $\sigma_{\mathrm{D},i} = 0$, his/her total resource extraction rate $R^*_{\mathrm{H},i}$ at equilibrium can be calculated analytically, by integrating the equilibrium extraction-rate density $H^*_i(x,y)$ over the entire exploitation kernel,

$$R^*_{\mathrm{H},i} = \int_0^L \int_0^L r_{\mathrm{H},i} R^*(x,y) E(x - x_i, y - y_i)\, \mathrm{d}x\mathrm{d}y, \tag{9}$$

where we have assumed that the exploitation kernel of the focal consumer does not overlap with the exploitation kernels of other consumers (we evaluate the accuracy of this approximation in Section S2.3). The equilibrium resource abundance can be calculated by setting $\mathrm{d}R(x,y)/\mathrm{d}t = 0$, giving

$$R^*(x,y) = K(1 - r_{\mathrm{H},i} E(x - x_i, y - y_i)/r). \tag{10}$$

Substituting E and R^* into Eq. 9, we get

$$R^*_{\mathrm{H},i} = 2\pi K \sigma_{\mathrm{H}}^2 r_{\mathrm{H},i} \left(\frac{1}{4} - \frac{r_{\mathrm{H},i}}{6r}\right). \tag{11}$$

To determine the sedentary yield-maximizing harvesting rate, we solve $\mathrm{d}R^*_{\mathrm{H},i}/\mathrm{d}r_{\mathrm{H},i} = 0$ for $r_{\mathrm{H},i}$ and find that this rate equals

$$r_{\mathrm{H,\,sedentary\text{-}yield\text{-}maximizing}} = \frac{3}{4} r. \tag{12}$$

The payoff to a sedentary consumer i is

$$\begin{aligned} V_i &= b_{\mathrm{H}} R^*_{\mathrm{H},i} - c_{\mathrm{H}} r^2_{\mathrm{H},i} \\ &= 2\pi b_{\mathrm{H}} K \sigma_{\mathrm{H}}^2 r_{\mathrm{H},i} \left(\frac{1}{4} - \frac{r_{\mathrm{H},i}}{6r}\right) - c_{\mathrm{H}} r^2_{\mathrm{H},i}. \end{aligned} \tag{13}$$

To determine the sedentary profit-maximizing harvesting rate, we set $\mathrm{d}V_i/\mathrm{d}r_{\mathrm{H},i} = 0$ for $r_{\mathrm{H},i}$ and find that this rate equals

$$r_{\text{H, sedentary-profit-maximizing}} = \frac{3}{4} r / (1 + \frac{6 r c_{\text{H}}}{2\pi K b_{\text{H}} \sigma_{\text{H}}^2}). \qquad (14)$$

The sedentary profit-maximizing harvesting rate is thus always smaller than the sedentary yield-maximizing harvesting rate and approaches the latter when the carrying-capacity density K, the harvesting efficiency $b_{\text{H}}/c_{\text{H}}$, or the harvesting radius σ_{H} are large.

S2.3 Effects of kernel overlap on the optimal strategies of sedentary consumers

Whereas mobile consumers can move to avoid overlap of other consumers' exploitation kernels with their own, sedentary consumers are more limited in this regard. Therefore, we test the effect of overlapping exploitation kernels on the optimal strategies of sedentary consumers by comparing the analytically calculated yield-maximizing harvesting rates and resource extraction rates (Eqs. 11 and 12, respectively, which assume that exploitation kernels do not overlap) with those obtained numerically (Figures S2E and S2F, respectively, which are calculated with consumers located at random locations in space and allowing kernel overlap).

The resource extraction rate of sedentary consumers initially increases with their harvesting rate, reaches a maximum at the yield-maximizing harvesting rate, and then declines with a further increase in harvesting rate (Figure S13A). Both the yield-maximizing harvesting rate and the yield-maximizing resource extraction rate decline with consumption density (orange line in Figure S13A) and deviate from the analytically calculated values (vertical and horizontal grey lines, respectively). To quantify these deviations, we examine how these rates drop relative to their analytical values as consumption density is increased (brown and orange lines in Figure S13B, respectively). And to elucidate how these drops are related to kernel overlap, we determine also the latter numerically (cyan line in Figure S13B). As expected, kernel overlap is small for low consumption densities, and the approximation accuracy is correspondingly high. While the approximation accuracy slowly declines with consumption density, it remains higher than 75% even for 50% consumption density, which is the consumption density used for Figures 4-7. At a consumption density of 100%, the effect of kernel overlap is more significant, causing a 40% decline in accuracy. Yet, it should be noticed that, when implementation errors in strategy imitation occur, even sedentary consumers have a small dispersal radius. This allows them to avoid kernel overlap with other consumers, and accordingly, the simplifying assumption of no kernel overlap describes them well.
