# Supplementary figures and images for: Emergence of social inequality in the spatial harvesting of renewable public goods
Source: PLoS Comput Biol. 2020 Jan 8;16(1):e1007483. doi: 10.1371/journal.pcbi.1007483 (PMC6974303; doi:10.1371/journal.pcbi.1007483)

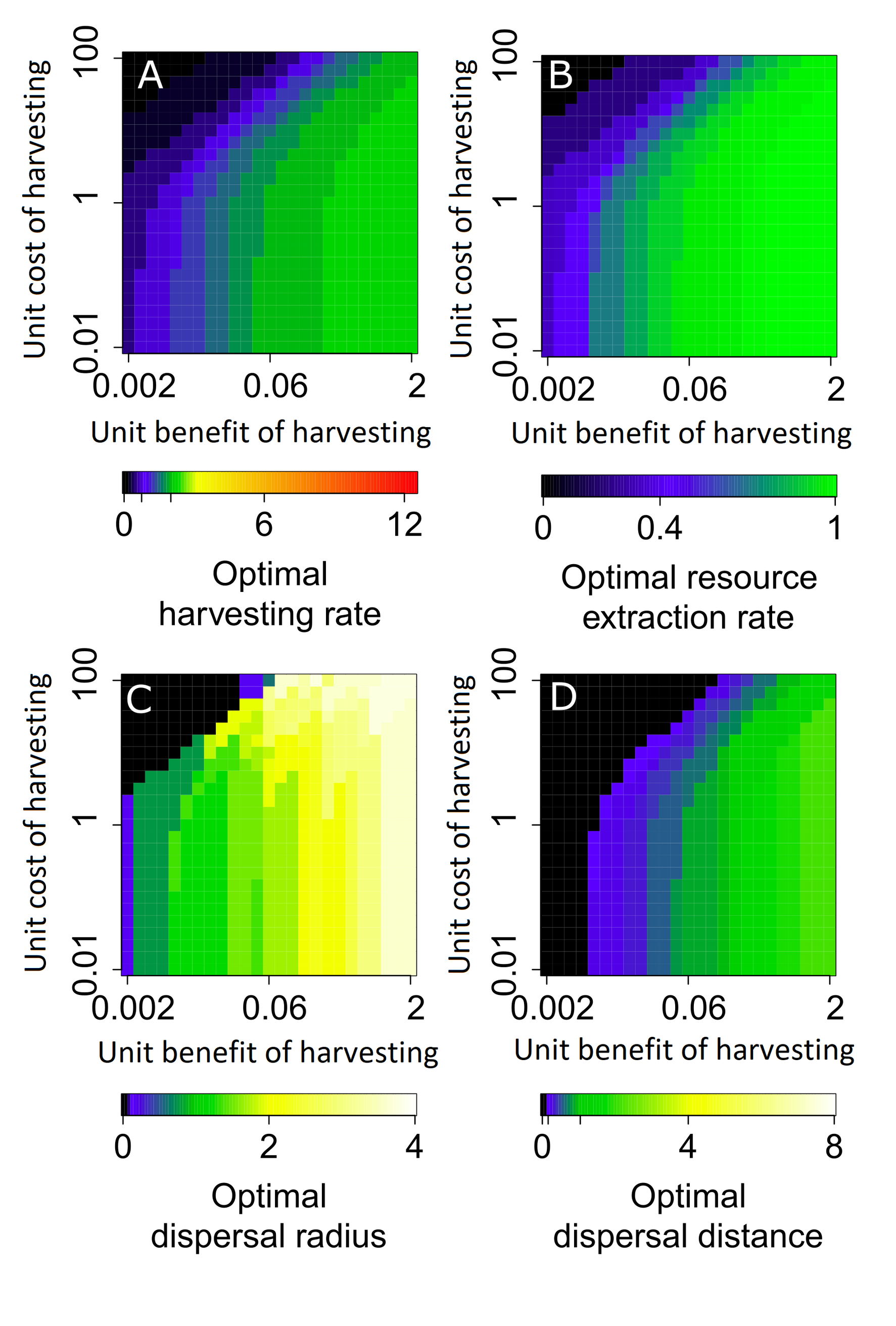

Supplement: S1 Fig — Effects of the unit benefit bH and cost cH of harvesting on the profit-maximizing (A) harvesting rates and (C) dispersal radii, and on the resultant (B) per capita resource extraction rates and (D) dispersal distances. In panel A, the sedentary and mobile yield-maximizing harvesting rates (S2 Appendix) are indicated, respectively, by the unlabeled tick marks in the blue and green ranges of the color bars. Comparing Figs 4B and 5A with panels A and B, respectively, shows that the evolved average harvesting rates and resource extraction rates are always lower than, respectively, the profit-maximizing harvesting rates and resource extraction rates, especially in the coexistence region, labeled ‘S & M’. Parameter values are as in Figs 4 and 5, while imitation parameters are not relevant to this figure. (TIF) [file pcbi.1007483.s005.tif]

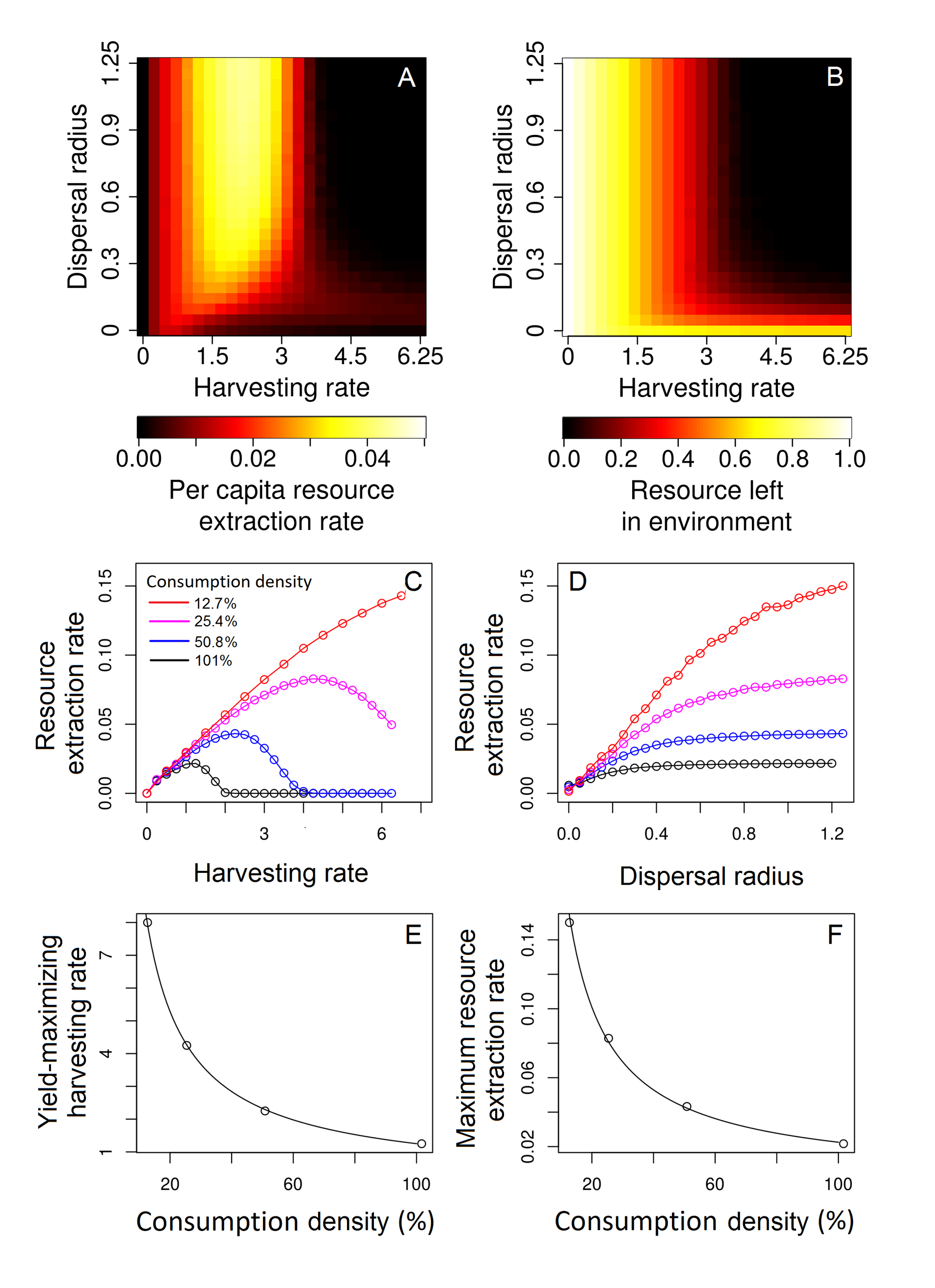

Supplement: S2 Fig — (A, B) Yield-maximizing resource-consumption strategies are found by computing and comparing the per capita resource extraction rates (expressed as fractions of the system’s carrying capacity extracted per unit time) of monomorphic populations with different harvesting rates and dispersal radii, here shown for a consumption density of 50.8% (expressed as the ratio of harvested area to total area). (C) The per capita resource extraction rate is maximized at an intermediate harvesting rate, called the yield-maximizing harvesting rate. (D) The resource-extraction rate increases monotonically with the dispersal radius, i.e., the yield-maximizing dispersal radius is infinite. (E-F) The yield-maximizing harvesting rate and the resultant maximum per capita resource extraction rate both decrease with consumption density. The maximum resource extraction rate is inversely proportional to the consumption density, with a power-law exponent close to –1. This indicates minimal kernel overlap among mobile consumers, i.e., mobile consumers evade through movement configurations in which their exploitation kernel overlaps with that of another consumer. Parameter values are as shown in Table 1, while imitation parameters are not relevant to this figure. (TIF) [file pcbi.1007483.s006.tif]

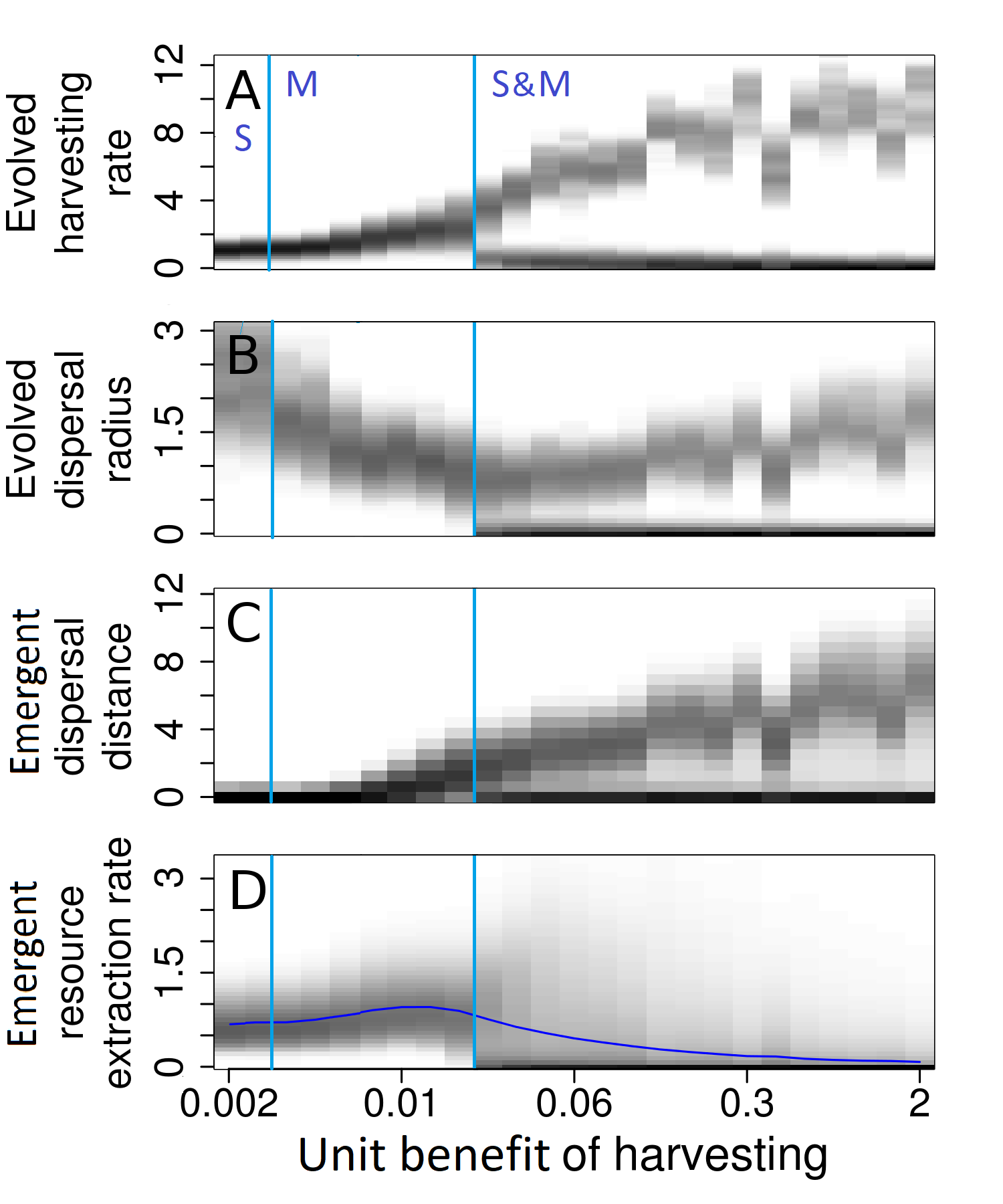

Supplement: S3 Fig — Effects of the unit benefit bH of harvesting on the evolved population distributions of (A) harvesting rates, (B) dispersal radii, (C) dispersal distances, and (D) per capita resource extraction rates. The blue curve in panel D shows the average per capita resource extraction rate. We can clearly see the three strategy regimes: sedentary (labelled ‘S’), mobile (labelled ‘M’), and coexistence of sedentary and mobile (labelled ‘S & M’). Parameter values are as in the row with cH = 0.68 in Fig 4 (Fig 4C and 4D show only dispersal distances and not also dispersal radii because the latter tend to drift in the region labeled ‘S,’ where the former are very small, as sustainable harvesting ensures that the resource densities at the locations of consumers very rarely fall below the dispersal threshold). (TIF) [file pcbi.1007483.s007.tif]

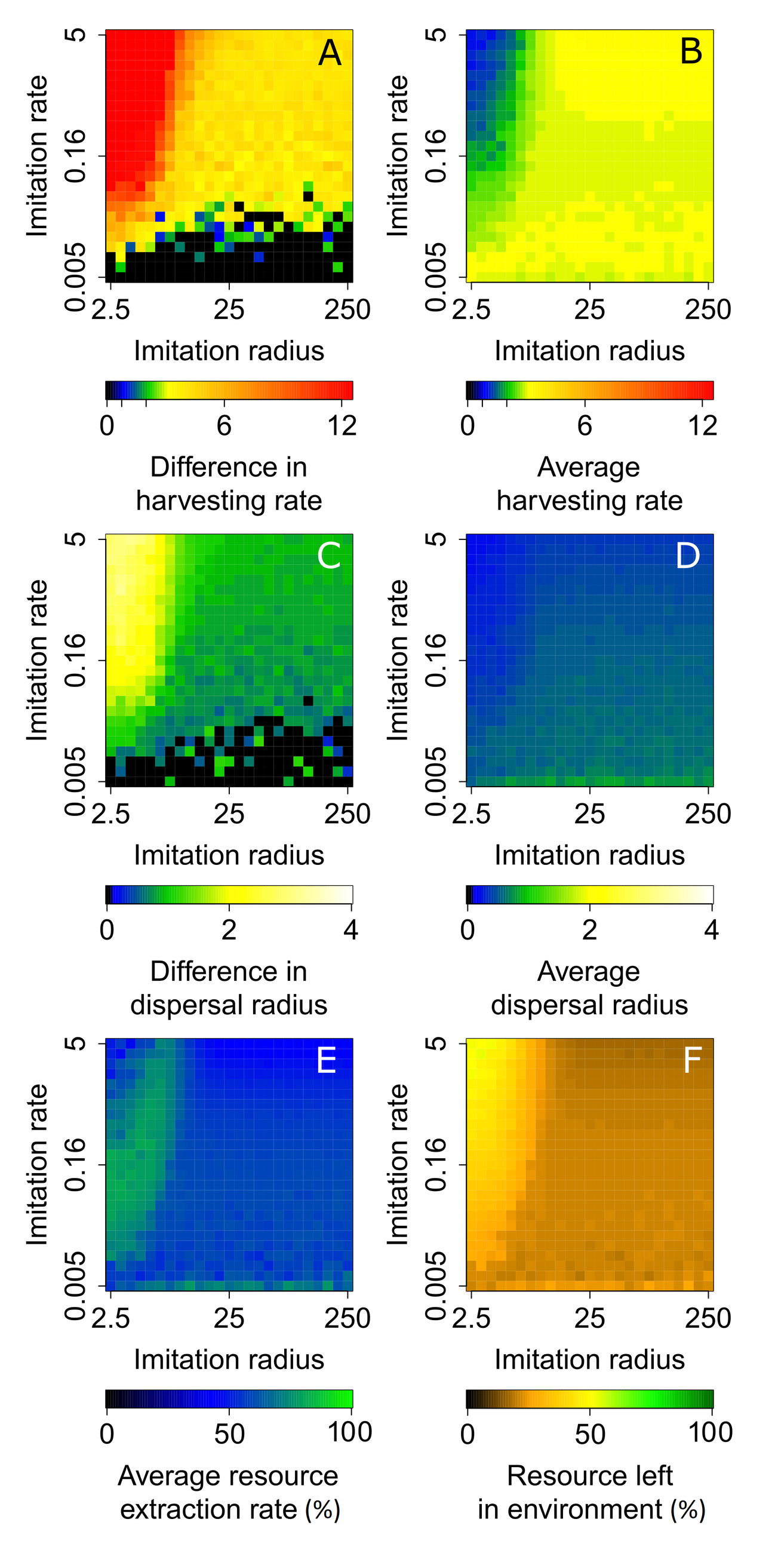

Supplement: S4 Fig — Effects of the imitation radius σI and imitation rate rI on the (A) difference between harvesting rates of sedentary and mobile consumers, (B) population average of harvesting rates, (C) difference between dispersal radii of sedentary and mobile consumers, (D) population average of dispersal radii, (E) average per capita resource extraction rate (expressed as a fraction of the yield-maximizing extraction rate), and (F) amount of resource left in the environment (expressed as a fraction of the system’s carrying capacity). While Figs 6 and 7 show how fast local imitation can lead to strategy diversification that does not occur for slow global imitation, here we show that this finding extends to parameter combinations in the coexistence region, where fast local imitation aggravates the aforementioned social inequality. For very low imitation rates (black regions in panels A and C), strategy diversification does not occur within the limited duration of the model runs considered here. Parameter values are as shown in Table 1. (TIF) [file pcbi.1007483.s008.tif]

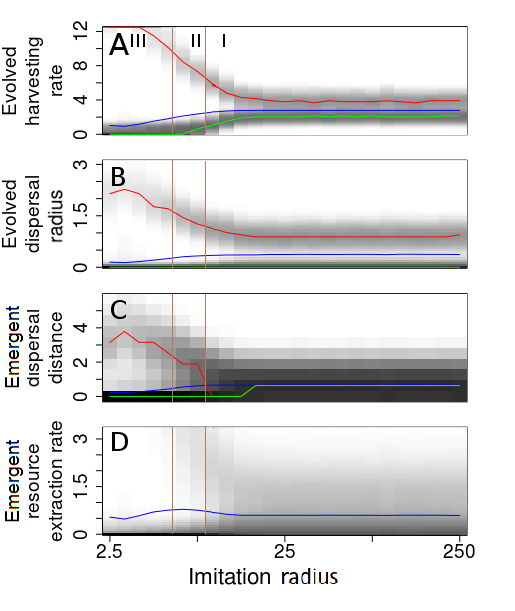

Supplement: S5 Fig — Effects of the imitation radius on the evolved population distributions of (A) harvesting rates, (B) dispersal radii, (C) dispersal distances, and (D) per capita resource extraction rates, for a large imitation rate corresponding to the top rows of panels in Fig 6. The blue curves in all panels show averages of the population distributions, while the red and green curves in panels A to C show averages of the population distributions separately for the two modes. We see that the per capita resource extraction rate is highest in the region labelled ‘II,’ in which the imitation radius is intermediate. For smaller imitation radii in the region labelled ‘III,’ the mobile consumers are very few but highly overexploitative, and therefore still drive the average resource extraction rate of the sedentary consumers to close to zero. For larger imitation radii in the region labelled ‘I,’ the sedentary consumers are also overexploitative, and the population’s average per capita resource extraction rate drops accordingly. Parameter values are as shown in Table 1, except for rI = 5. (TIF) [file pcbi.1007483.s009.tif]

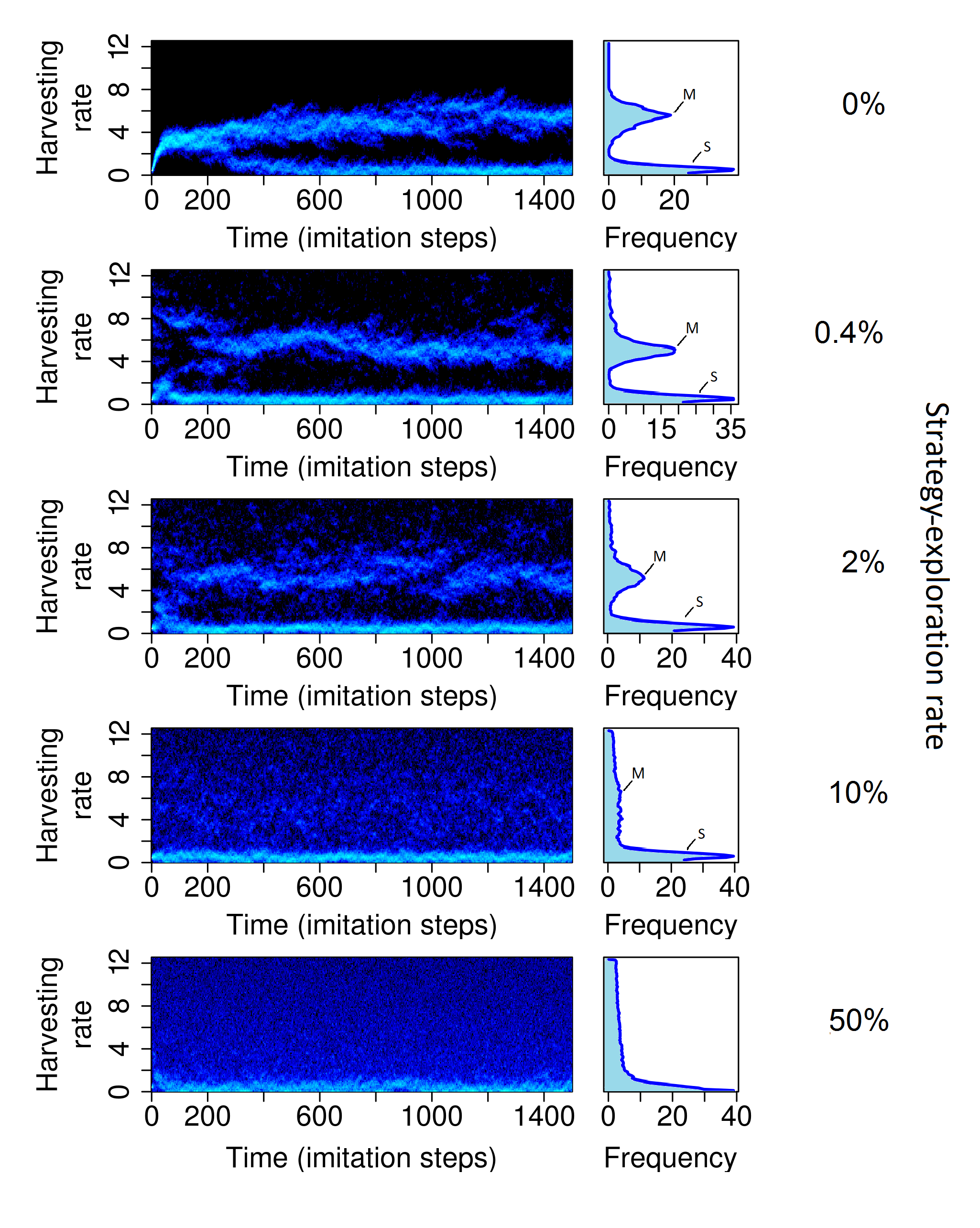

Supplement: S6 Fig — Time series of population distributions of harvesting rates for five different strategy-exploration rates shown in the rightmost column (expressed as fractions of the imitation rate), shown alongside the time-averaged population distributions (averaged over the last 1,000 units to exclude the initial transient), with the frequencies in the separate bins adding up to the number N of consumers. Upon an exploration event, consumers change their harvesting rates and dispersal radii to values randomly drawn from uniform distributions over the intervals [0, 12.5] and [0, 2.5], respectively. For strategy-exploration rates of up to about 2% of the imitation rate, the evolutionary outcome remains qualitatively unchanged, with the two modes corresponding to frugal sedentary consumers (labeled ‘S’) and overexploitative mobile consumers (labeled ‘M’), respectively. Higher strategy-exploration rates widen these modes to an extent that the separation between them is lost. Parameter values are as shown in Table 1, except for cH = 0.68. (TIF) [file pcbi.1007483.s010.tif]

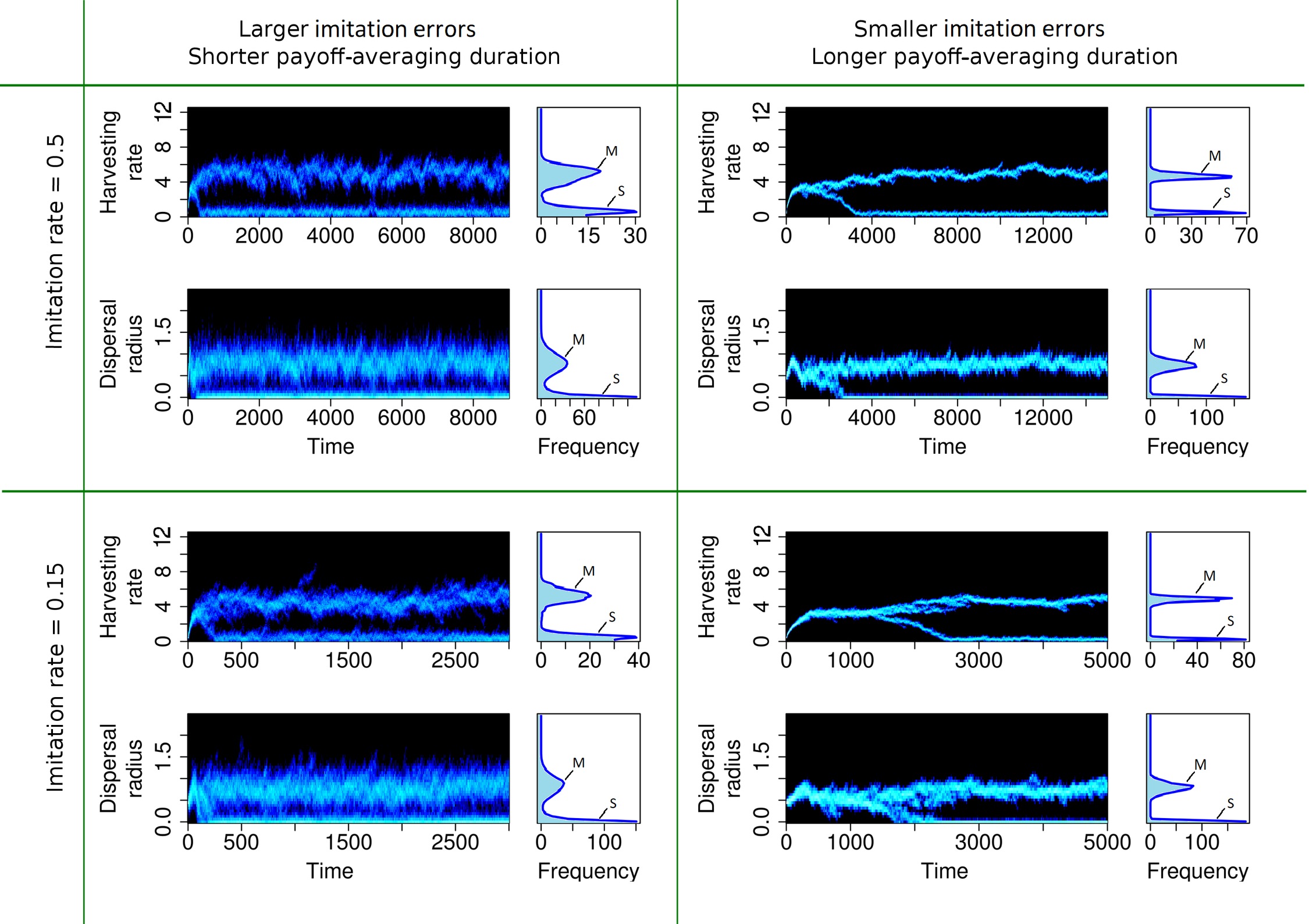

Supplement: S7 Fig — The left column shows time series of the population distributions and time-averaged distributions of harvesting rates and dispersal radii for the default parameter values, while the right column shows the same information for smaller imitation errors and longer payoff-averaging durations. These time series are shown alongside the time-averaged population distributions (averaged over the last one third of the total simulated duration to exclude the initial transient, with the frequencies in the separate bins adding up to the number N of consumers). The two rows show results for two different imitation rates, rI = 0.5 in the upper row and rI = 0.15 in the lower row. As expected, the evolutionary dynamics in the right column are slower and the resultant modes are narrower than in the left column. Nevertheless, the evolutionary dynamics in the two columns are analogous, exhibiting the same pattern of diversification into sedentary and mobile resource-consumption strategies and leading to roughly the same average harvesting rates and dispersal radii in the two modes. Parameter values are as shown in Table 1, except for T = 1, μH = 0.0125, and μD = 0.00125 in the right column. (TIF) [file pcbi.1007483.s011.tif]

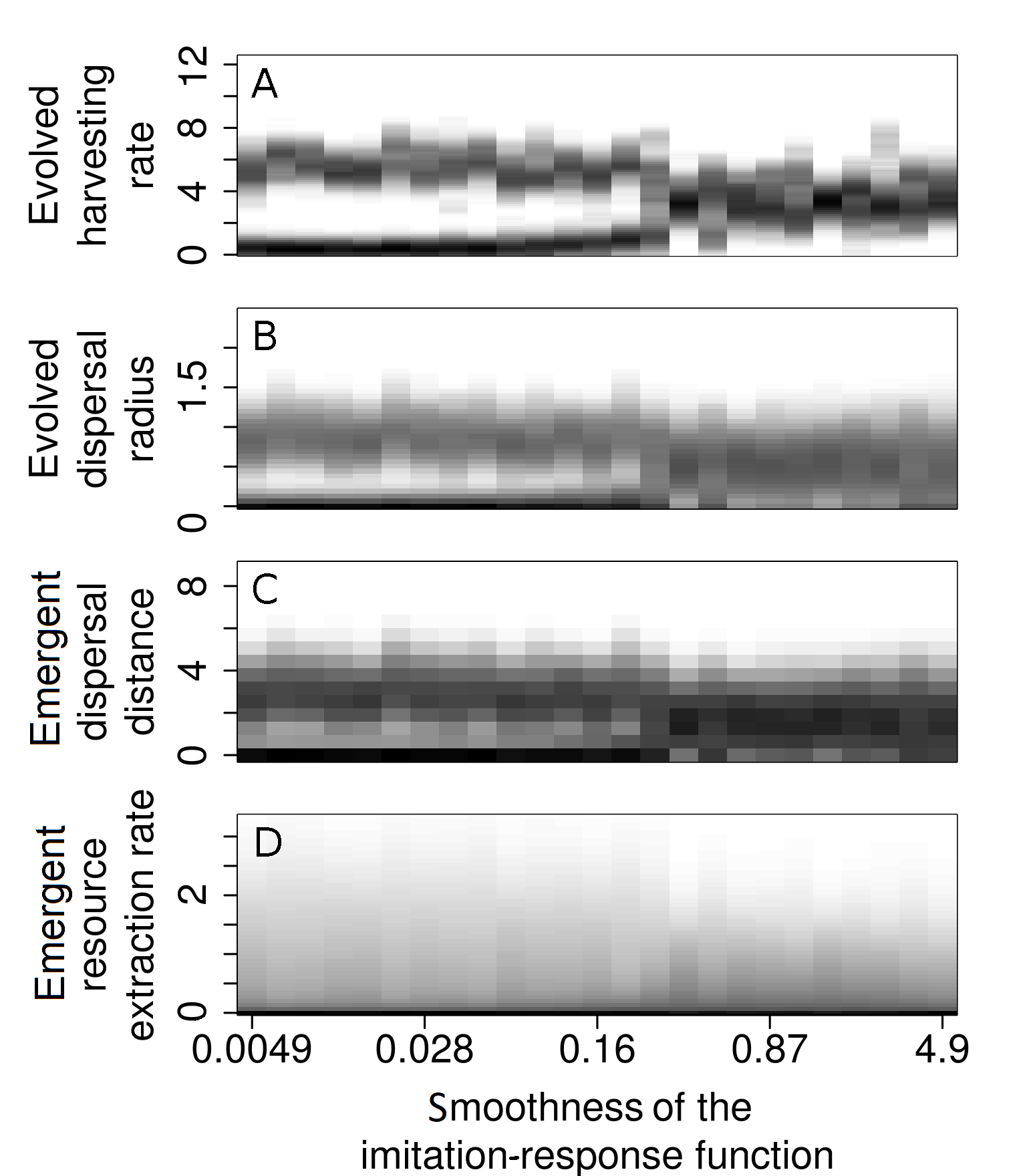

Supplement: S8 Fig — Effects of the smoothness of the imitation-response function on the evolved population distributions of (A) harvesting rates, (B) dispersal radii, (C) dispersal distances, and (D) per capita resource extraction rates. The smoothness is measured as wI/ΔV95, where the width wI is the range of payoff differences over which the imitation-response function changes from 5% to 95%, and ΔV95 is the 5% to 95% quantile range of the payoff differences experienced by consumers. Our results are robust until the aforementioned width exceeds about a tenth of the experienced payoff differences. Higher smoothness promotes unsustainable resource-consumption strategies and causes overexploitation of the resource. Parameter values are as shown in Table 1. (TIF) [file pcbi.1007483.s012.tif]

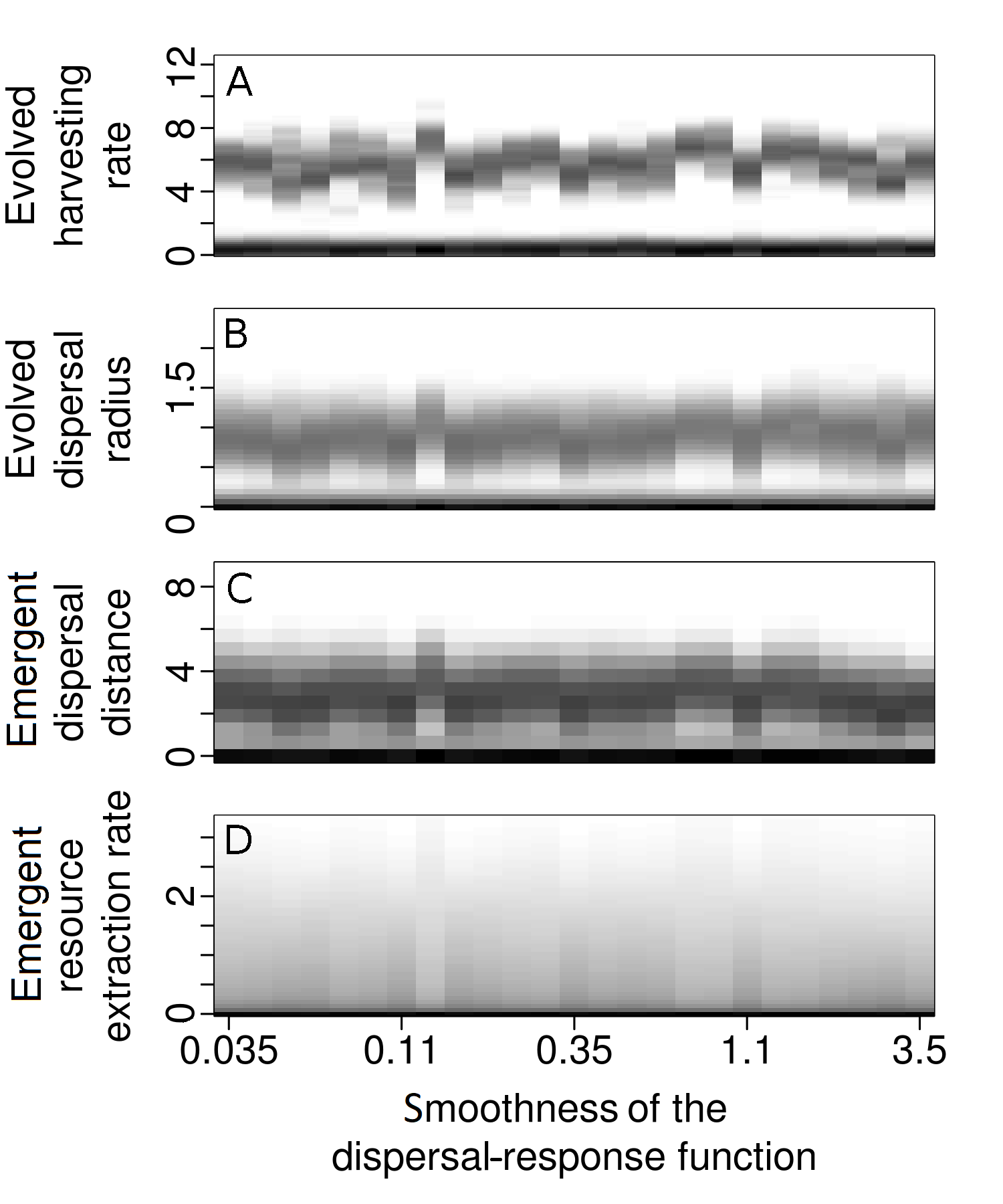

Supplement: S9 Fig — Effects of the smoothness of the dispersal-response function on the evolved population distributions of (A) harvesting rates, (B) dispersal radii, (C) dispersal distances, and (D) per capita resource extraction rates. The smoothness is measured as wD/ΔR95, where the width wD is the range of resource densities over which the dispersal-response function changes from 5% to 95%, and ΔR95 is the 5% to 95% quantile range of the resource densities experienced by consumers. Our results are robust over several orders of magnitude of the dispersal-response smoothness. Parameter values are as shown in Table 1. (TIF) [file pcbi.1007483.s013.tif]

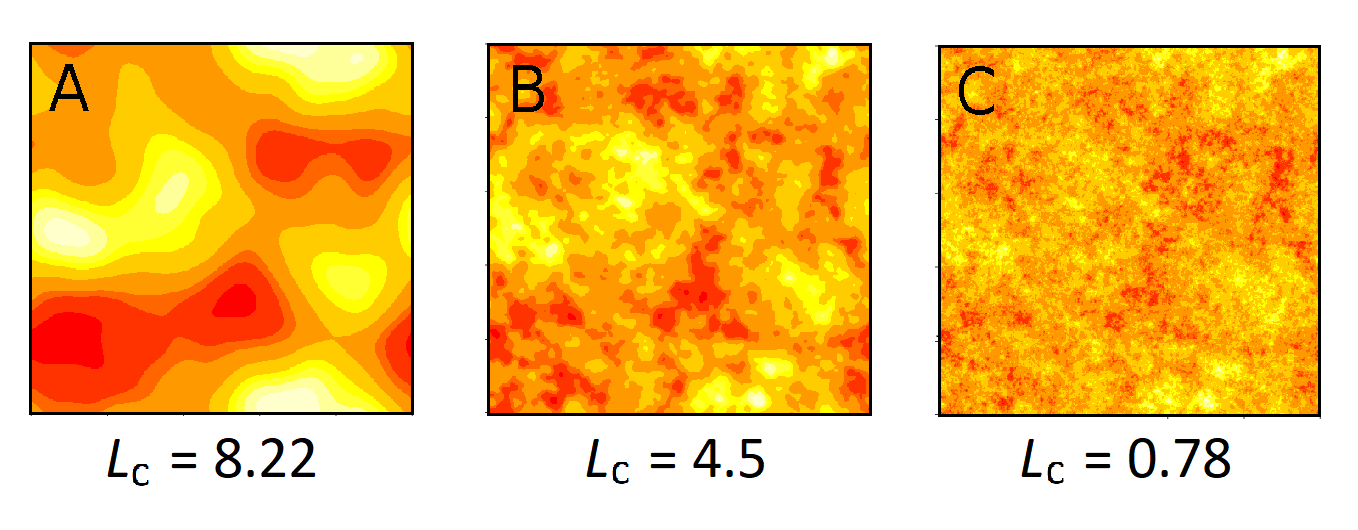

Supplement: S10 Fig — (A-C) Heterogeneous spatial distributions of the intrinsic resource growth rate with different spatial correlation lengths Lc. The shown spatial distributions are generated using a synthetic turbulence model [52], the code for which can be found at https://github.com/jaideep777/Consumer-resource-system/blob/master/src/turbulence.cu. (TIF) [file pcbi.1007483.s014.tif]

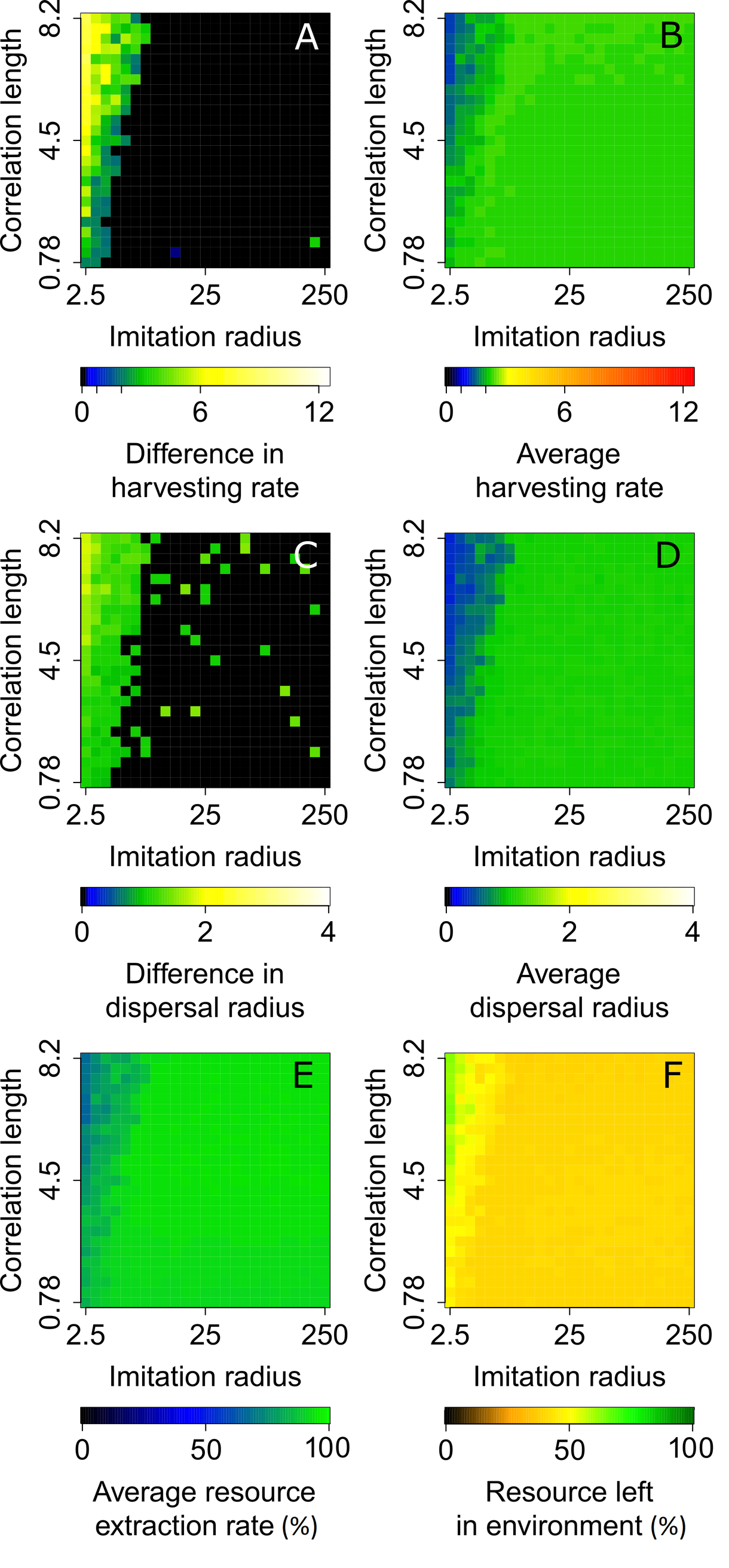

Supplement: S11 Fig — Effects of the imitation radius σI and correlation length Lc of heterogeneous spatial distributions of the intrinsic resource growth rate on the (A) difference between harvesting rates of sedentary and mobile consumers, (B) population average of harvesting rates, (C) difference between dispersal radii of sedentary and mobile consumers, (D) population average of dispersal radii, (E) average per capita resource extraction rate (expressed as a fraction of the yield-maximizing extraction rate), and (F) amount of resource left in the environment (expressed as a fraction of the system’s carrying capacity). For fast imitation, spatial heterogeneity causes strategy diversification roughly when the correlation length Lc exceeds the imitation radius σI. Parameter values are as shown in Table 1, except for bH =0.14, cH = 0.8, and rI = 0.5. (TIF) [file pcbi.1007483.s015.tif]

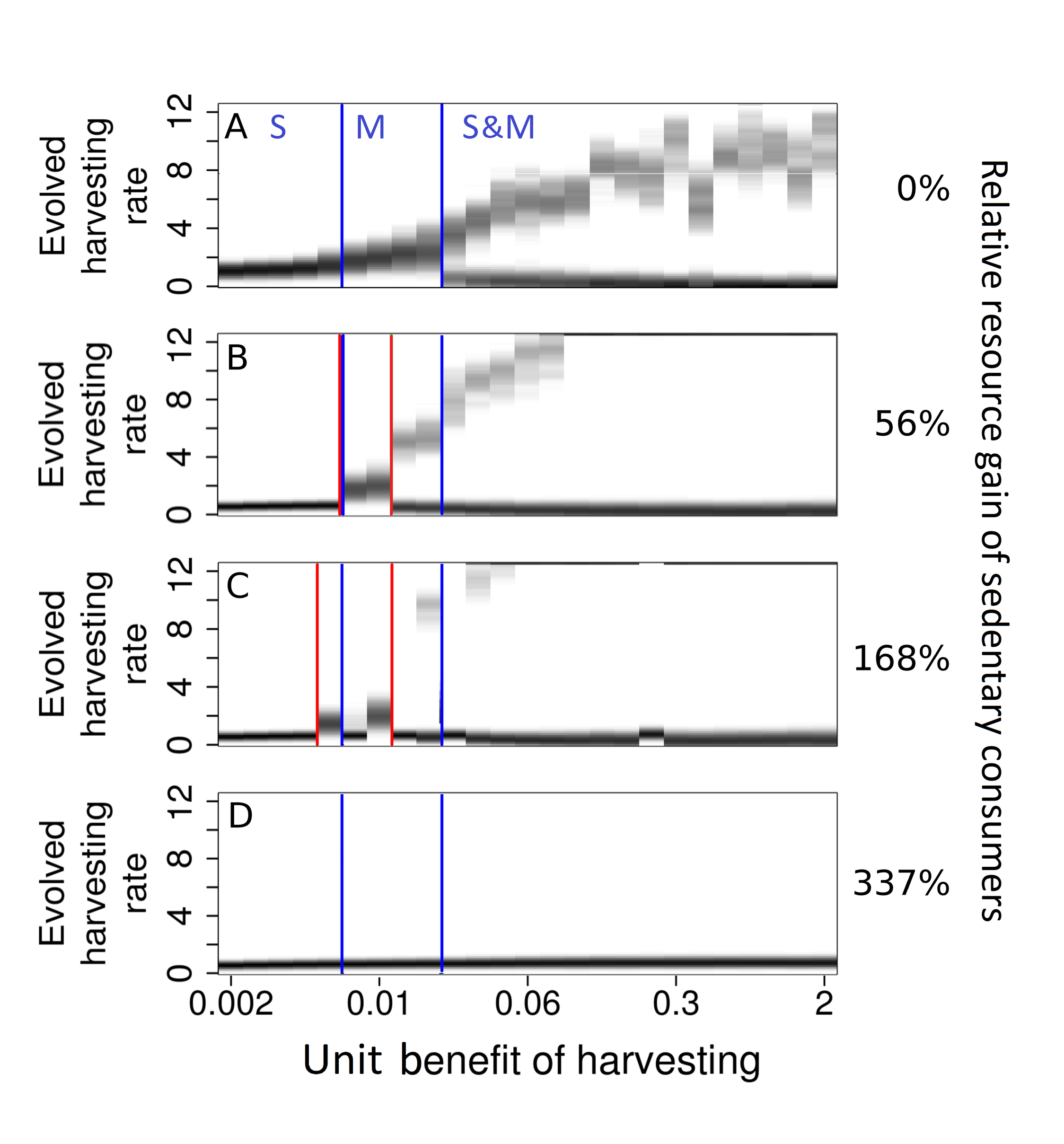

Supplement: S12 Fig — Effects of the unit benefit bH of harvesting on the evolved population distributions of harvesting rates for four different values of RG, the fixed additional supply of resource per unit time available to consumers who do not disperse, shown in the rightmost column (expressed as fractions of the maximum resource extraction rate of sedentary consumers shown in Equation 11). (A) For RG = 0, the three strategy regimes shown in S3 Fig apply: sedentary (labelled ‘S’), mobile (labelled ‘M’), and coexistence of sedentary and mobile (labelled ‘S & M’). (B, C) Cases with RG>0 describe scenarios in which sedentary resource consumption enables increased ecological efficiency (e.g., through agricultural activities) or decreased competition with mobile consumers (e.g., through diet specialization). To offset the additional resource supply of the sedentary consumers, the mobile consumers harvest at higher rates, which enables them to keep coexisting with the sedentary consumers. (D) Only when the additional resource supply of the sedentary consumers is much greater than what is otherwise available to them, sedentary consumers outcompete mobile consumers. The red lines indicate how the boundaries between the three strategy regimes shift as RG is raised from 0. Parameter values are as in S3 Fig. (TIF) [file pcbi.1007483.s016.tif]

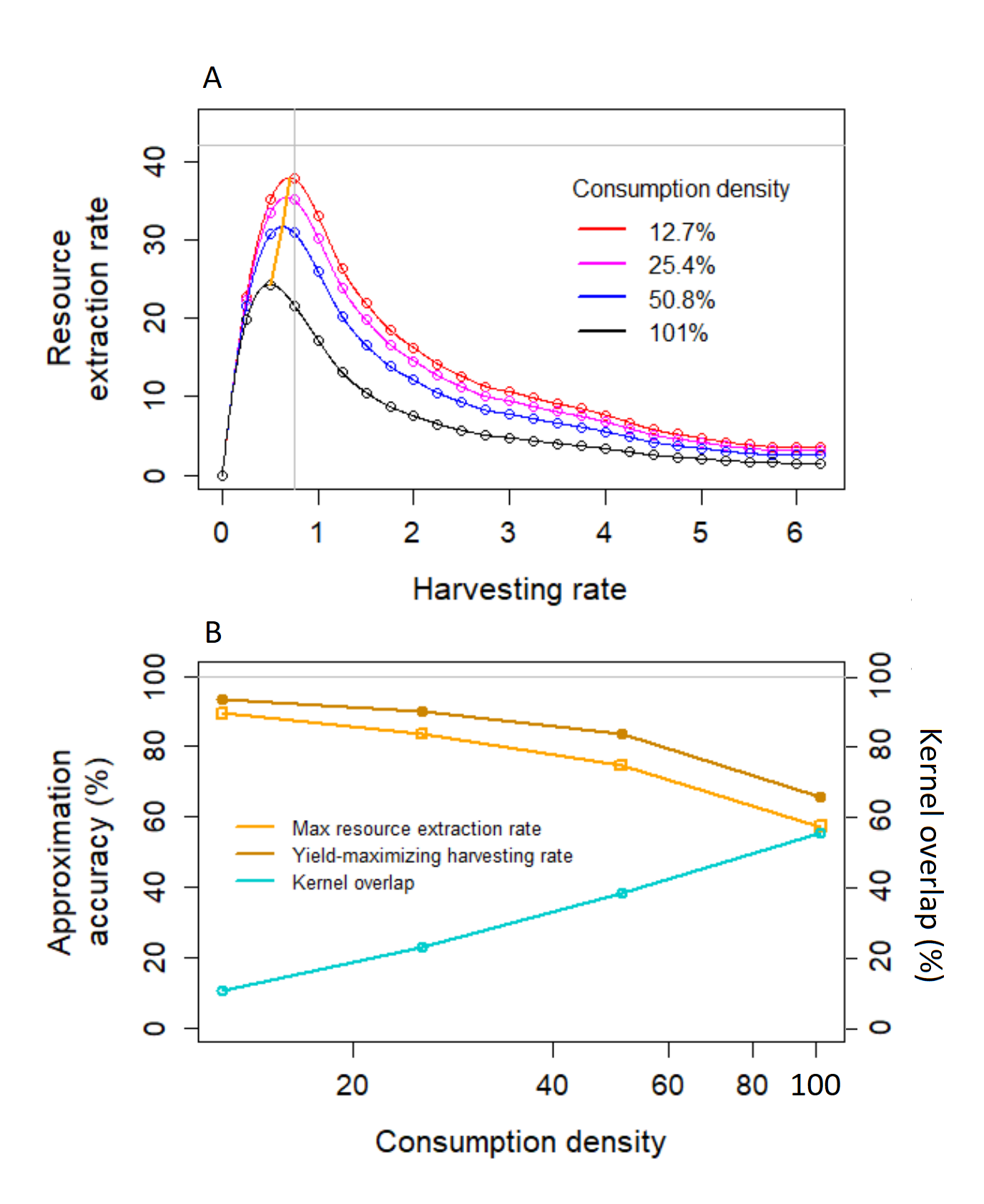

Supplement: S13 Fig — (A) Maximum resource extraction rates of randomly placed sedentary consumers as a function of their harvesting rate, for four different consumption densities (red, magenta, blue, and black circles with fitted cubic splines). The orange line indicates the numerically calculated yield-maximizing harvesting rates (horizontal coordinates along orange line) and maximum resource extraction rates (vertical coordinates along orange line). These are compared with the corresponding analytical values (horizontal and vertical grey lines, respectively), calculated assuming no overlap of exploitation kernels (S2 Appendix). (B) Approximation accuracy, defined as the percentage drop in the maximum resource extraction rate (orange line) and the yield-maximizing harvesting rate (brown line) relative to their respective analytical values, as a function of consumption density. At low consumption densities, the maximum resource extraction rate and the yield-maximizing harvesting rate are both close to their respective analytical values, indicating that the effects of kernel overlap (cyan line) are small. The approximation accuracy remains higher than 75% even at 50% consumption density and decreases to no less than 60% at 100% consumption density. The relative kernel overlap is defined as the average fraction of a consumer’s exploitation area that overlaps with that of other consumers. Accordingly, the relative drop in maximum resource extraction rate mirrors the relative kernel overlap. (TIF) [file pcbi.1007483.s017.tif]
